# Supplementary material for: The Pan-African Surgical Healthcare Forum: An African qualitative consensus propagating continental national surgical healthcare policies and plans
Source: PLOS Glob Public Health. 2024 Nov 12;4(11):e0003635. doi: 10.1371/journal.pgph.0003635 (PMC11556714; doi:10.1371/journal.pgph.0003635)
Supplement: S2 Appendix — (PDF) [file pgph.0003635.s003.pdf]

S2 Appendix. Pan-African Surgical Healthcare Forum Plenary Sessions and Facilitators

	Topic	Facilitators
1	Opening Plenary	Honourable Minister Dr Sabin Nsanzimana, Ministry of Health, Rwanda<br>Professor Abebe Bekele, Co-Chair, Center for Equity in Global Surgery; Dean, University of Global Health Equity<br>Dr Susannah Schaefer, President, Smile Train<br>Mrs Nkeiruka Obi, Vice President and Regional Director Africa, Smile Train
2	The History of the World Health Assembly Resolution 68.15 and national Surgical Obstetrics and Anesthesia Plans	Professor Emmanuel M. Makasa, Adjunct Professor of Global Surgery, Founding Director SADC WitSSurg
3	What are National Surgical Plans/Policy?	Professor Emmanuel A. Ameh, National Hospital, Abuja, Nigeria
4	National Surgical Plans: Global status with particular emphasis on the African continent	Professor Abebe Bekele, Co-Chair, Center for Equity in Global Surgery; Dean, University of Global Health Equity
5	Experience from Ethiopia	Dr Elubabor Buno, Lead Executive Officer of Medical Services, Ministry of Health- Ethiopia
6	Experience from Zambia	Dr Christopher Chanda, National Coordinator of Anaesthesia, Ministry of Health- Zambia
7	Experience from Rwanda	Dr Parfait Uwaliraye, Head of Planning, M&E and Health Financing Department, Ministry of Health – Rwanda
8	Experience from Nigeria	Dr Bitrus Oghoghorie Deborah, Consultant Grade 1, Federal Ministry of Health, Hospital Services- Nigeria
9	Experience from Zimbabwe	Shingai Nyaguse-Chiurunge, Consultant anesthesiologist, President Zim Senior Hospital Doctors Association-Zimbabwe
10	Experience from Madagascar	Dr Rado Razafimahatratra, Ministry of Public Health-Madagascar
11	Experience from Namibia	Francina Marukuavi Ngakuzevi, Ministry of Health- Namibia
12	Experience from Angola	State Minister for Health, Angola

13	The Process of National Surgical Policy/Plan Development	Dr Faustin Ntirenganya, Head of Surgery, School of Medicine and Pharmacy, College of Medicine and Health Sciences, University of Rwanda.
14	The Roadmap to National Surgical Obstetrics and Anaesthesia Plan Implementation	Professor Emmanuel M. Makasa, Adjunct Professor of Global Surgery, Founding Director SADC WitSSurg
15	Role of Local and Global Partners in National Surgical Obstetrics and Anaesthesia Plan Development	Dr Robert Riviello, Co-Chair, Center for Equity in Global Surgery; Head of Surgery, University of Global Health Equity; Director, Harvard Program in Global Surgery and Social Change
16	Funding in Global Surgery	Professor Emmanuel A. Ameh, National Hospital, Abuja, Nigeria
17	Advocacy in Global Surgery	Dr Justina Seyi-Olajide, Lagos University Teaching Hospital, Lagos, Nigeria
