# Supplementary material for: The Pan-African Surgical Healthcare Forum: An African qualitative consensus propagating continental national surgical healthcare policies and plans
Source: PLOS Glob Public Health. 2024 Nov 12;4(11):e0003635. doi: 10.1371/journal.pgph.0003635 (PMC11556714; doi:10.1371/journal.pgph.0003635)
Supplement: S3 Appendix — (PDF) [file pgph.0003635.s004.pdf]

S3 Appendix. English, French, Portuguese, and Spanish PASHeF 2023 Consensus Statement

English

PASHeF 2023 Consensus Statement

1. Country delegates unanimously agreed that national surgical plans/policies are important to Africa.

2. Country delegates agreed that all African countries should complete their plans/policies at the soonest.

3. Country delegates recognized that the building blocks of the plan/policy can be modified to address each country's needs.

4. There was emphasis on "not being married" to the acronym NSOAP, as it may result in lack of buy-in from some key stakeholders.

5. Country delegates agreed that surgical plan/policy implementation can lead to National Health Systems Strengthening.

6. Country delegates urged all countries to accelerate progress towards UHC and the SDGs to meet the 2030 goals as specified in the WHA resolution 68.15.

7. Countries need to urgently implement schemes for health insurance that covers all surgical conditions.

8. The leadership of a "Surgical plan/policy champion" would add value to the planning/policy-making and implementation.

9. However, all country delegates cautioned NSOAPs alone are not the ultimate solutions to the lack of access to surgical care in the continent.

10. Surgical plan/policy serves as the foundation, but implementation of the plan/policy is critical.

PASHeF 2023 Consensus Statement

11. Governments should commit to improve the surgical infrastructure and equipment and supply chain structure in their countries as part of plan/policy implementation.

12. Country delegates recommended surgical plans/policies should be designed to serve as integral components of the existing health care policy framework in countries.

13. Country delegates agreed that surgical plan/policy development and implementation should be based on local solid evidence and data (from district level to national level).

14. They also recommended countries should strengthen and use their national health information systems and other healthcare data sources to capture surgical data.

15. Countries should have a clear surgical care monitoring and evaluation tools, and sets of indicators.

16. Country delegates recognized the following opportunities in surgical plan/policy development and implementation: regional partnerships for sharing of resources and expertise; existing success stories in surgical planning/policy-making in African countries; a young and energetic workforce in African countries.

17. Country delegates identified the following barriers to surgical plan/policy development and implementation: inadequate financing for planning/policy-making and implementation; reduced political will and governmental ownership; competing priorities at a national and local level; human resource issues (lack of expertise and motivation resulting in brain drain); the systematic exclusion of surgical care in the last few decades from primary healthcare; shortage of resources; insufficient technical equipment and

biomedical support; disparity between written budgets and actual financing of surgical care.

18. Country delegates agreed the proposed steps of National Surgical Healthcare Policy development are appropriate.

19. Country delegates agreed that the process should also include the community (end user) in planning, implementation and leadership.

20. Country delegates concurred that Policies should focus on the entire spectrum of surgical care (prevention, perioperative care, and rehabilitation).

21. Plans/policies should also emphasize capacity building, quality of care, access to care and sustainability.

22. Country delegates proposed the creation of a Pan-African/regional Surgical Healthcare Policy monitoring system composed of member states to follow up the continental progress.

23. Monitoring and evaluation need to be aligned with Quality Assurance.

24. Countries encouraged the creation of a dedicated surgical care leadership department/directorate at the MoH level with dedicated and qualified professionals.

25. Members want to see surgical plans/policies institutionalised within the national healthcare systems to allow for continuity.

26. Countries should bring stakeholder sector ministries into an inter-ministerial task force for joint leadership and supportive supervision.

27. Country delegates recommended this plan/policy should be embraced at the highest level of government (Cabinet and Parliament).

PASHeF 2023 Consensus Statement

28. MoHs should create a committee (TWG) or advisory council of experts to advise and monitor the implementation of the plans/policies. The leadership structure should also be reflected at the district level.

29. WHO should be an integral part of the planning/policy-making and implementation, in close partnership with the MoHs.

30. Country delegates recommended Ministries of Health should establish a budget line specific to Surgical and Anesthesia care.

31. Budgets for surgical care should be managed at every level – MoH, Hospitals, Health Center. Budget should be dynamic and incremental.

32. The national essential drug and resources list should include the appropriate surgical equipment and consumables.

33. Countries should implement innovative ways of surgical healthcare financing.

34. Countries should engage the private sector to mobilise resources for surgical system strengthening

35. Country delegates identified critical stakeholders in the process of Surgical Healthcare Plan/Policy development: including local governmental level leadership (MoH, Ministries of Finance); end users (patient groups and the community) such as women groups; the private sector and industry; professional societies; academia; NGOs; the military; WHO and other global bodies; African Union; funding agencies such as World Bank, IMF, AfDB; regional bodies such as the SADC, EAC, WAHO, ECOWAS, CEMAC and COMESA among others.

36. MoHs should strengthen their partnerships with international organisations, regional bodies and development partners.

PASHeF 2023 Consensus Statement

37. Partnerships should be equitable and transparent.

38. Stakeholders should be engaged early in the process of Surgical Healthcare Plan/Policy development.

39. Country delegates underscored the value of celebrities as advocates for surgical planning/policy-making.

40. All country delegates agreed that community level advocacy are essential to promote prevention of surgical diseases.

41. The role of the media as a positive influencer of Surgical Healthcare Plans/Policies is key for the implementation of African Surgical Healthcare Plans/Policies.

42. Surgical systems improvement should be advanced as a public health concern.

43. Country delegates recommended the following: facilitated and fast-track importation of equipment; taxation-free importation of surgical consumables and equipment; regional harmonization of training curricula; unified regional procurement system; standardization of care delivery guidelines and protocols; strengthen biomedical and infrastructure maintenance.

44. PASHeF should be an annual platform for countries to share experience, request for technical exchange and support, and engage global partners.

45. PASHeF should invite other global partners to the annual sessions.

46. PASHeF should be hosted by alternating countries.

47. Each African country should have a focal person represented at the PASHeF.

PASHeF 2023 Consensus Statement

48. PASHeF should develop an inclusive governance system that is representative of the whole continent.

49. All country delegates requested this consensus document to be formally submitted to their Ministers of Health for accelerated implementation.

50. All country delegates recommended that issues of surgical healthcare should be an agenda item on ministerial meetings, both at the African Union meetings and sub-regional meetings.

French

Déclaration de consensus PASHeF 2023

1. Les délégués des pays ont unanimement convenu que les plans/politiques chirurgicaux nationaux sont importants pour l'Afrique.

2. Les délégués des pays ont convenu que tous les pays africains devraient achever leurs plans/politiques dans les meilleurs délais.

3. Les délégués des pays ont reconnu que les éléments constitutifs du plan/de la politique peuvent être modifiés pour répondre aux besoins de chaque pays.

4. Il a été souligné l'importance de ne pas être trop attaché à l'acronyme NSOAP, car cela peut entraîner un manque d'adhésion de la part de certains acteurs clés.

5. Les délégués des pays ont convenu que la mise en œuvre de plans/politiques chirurgicaux peut conduire au renforcement des systèmes de santé nationaux.

6. Les délégués des pays ont exhorté tous les pays à accélérer les progrès vers la couverture sanitaire Universelle (CSU) et les objectifs de Développement Durable (ODD) afin d'atteindre les objectifs de 2030, comme spécifié dans la résolution 68.15 de l'Assemblée Mondiale de la Santé.

7. Les pays doivent mettre en place de toute urgence des régimes d'assurance maladie couvrant toutes les affections chirurgicales.

8. La direction d'un "champion du plan/politique chirurgicale" apporterait une valeur ajoutée à la planification, à l'élaboration des politiques et à leur mise en œuvre.

Déclaration de consensus PASHeF 2023

9. Cependant, tous les délégués des pays ont mis en garde en soulignant que les NSOAPs (Plans nationaux de chirurgie, d'obstétrique et d'anesthésie) ne constituent pas en eux-mêmes la solution ultime au manque d'accès aux soins chirurgicaux sur le continent.

10. Le plan/la politique chirurgicale sert de base, mais la mise en œuvre du plan/de la politique est essentielle.

11. Les gouvernements devraient s'engager à améliorer l'infrastructure chirurgicale, l'équipement et la structure de la chaîne d'approvisionnement dans leurs pays, dans le cadre de la mise en œuvre des plans/politiques.

12. Les délégués des pays ont recommandé que les plans/politiques chirurgicaux soient conçus pour faire partie intégrante du cadre politique de soins de santé existant dans les pays.

13. Les délégués des pays ont convenu que l'élaboration et la mise en œuvre de plans/politiques chirurgicaux devraient être fondées sur des preuves et des données solides locales (du niveau du district au niveau national).

14. Ils ont également recommandé aux pays de renforcer et d'utiliser leurs systèmes nationaux d'information sanitaire et d'autres sources de données sur les soins de santé pour saisir les données chirurgicales.

15. Les pays doivent disposer d'outils clairs de suivi et d'évaluation des soins chirurgicaux et d'ensembles d'indicateurs.

16. Les délégués des pays ont reconnu les opportunités suivantes dans l'élaboration et la mise en œuvre de plans/politiques chirurgicaux : partenariats régionaux pour le partage des ressources et de l'expertise ; les réussites existantes en matière de planification/élaboration de politiques chirurgicales dans les pays africains ; une main-d'œuvre jeune et dynamique dans les pays africains.

Déclaration de consensus PASHeF 2023

17. Les délégués des pays ont identifié les obstacles suivants à l'élaboration et à la mise en œuvre des plans/politiques chirurgicaux : un financement insuffisant pour la planification/l'élaboration et la mise en œuvre des politiques ; une volonté politique et une appropriation gouvernementale réduites ; des priorités concurrentes aux niveaux national et local ; problèmes de ressources humaines (manque d'expertise et de motivation entraînant une fuite des cerveaux) ; l'exclusion systématique des soins chirurgicaux au cours des dernières décennies des soins de santé primaires ; une pénurie de ressources; un équipement technique et un support biomédical insuffisants ; et disparité entre les budgets écrits et le financement réel des soins chirurgicaux.

18. Les délégués des pays ont convenu que les étapes proposées pour l'élaboration de la politique nationale de soins chirurgicaux étaient appropriées.

19. Les délégués des pays ont convenu que le processus devrait également inclure la communauté (bénéficiaire de services) dans la planification, la mise en œuvre et le leadership.

20. Les délégués des pays ont convenu que les politiques devraient se concentrer sur l'ensemble du spectre des soins chirurgicaux (prévention, soins périopératoires et réadaptation).

21. Les plans/politiques doivent également mettre l'accent sur le renforcement des capacités, la qualité des soins, l'accès aux soins et la durabilité.

22. Les délégués des pays ont proposé la création d'un système de suivi de la politique de soins de santé chirurgicaux pan-africain/régional, composé d'États membres, afin de suivre les progrès continentaux.

23. Le suivi et l'évaluation doivent être alignés sur l'assurance de qualité.

Déclaration de consensus PASHeF 2023

24. Les pays ont encouragé la création d'un département/direction dédié à la direction des soins chirurgicaux au niveau du ministère de la Santé avec des professionnels dévoués et qualifiés.

25. Les membres souhaitent voir les plans/politiques chirurgicaux institutionnalisés au sein des systèmes de santé nationaux pour permettre la continuité.

26. Les pays doivent intégrer les ministères sectoriels concernés dans un groupe de travail interministériel pour un leadership conjoint et une supervision de soutien.

27. Les délégués des pays ont recommandé que ce plan/politique soit adopté au plus haut niveau du gouvernement (Cabinet et Parlement).

28. Les ministères de la santé doivent créer un groupe de Travail Technique (GTT) ou un conseil consultatif d'experts pour conseiller et surveiller la mise en œuvre des plans/politiques. La structure de direction doit également être reflétée au niveau du district.

29. L'OMS devrait être une partie intégrante de la planification, de l'élaboration des politiques et de leur mise en œuvre, en étroite collaboration avec les ministères de la Santé.

30. Les délégués des pays ont recommandé que les ministères de la Santé établissent un budget spécifique pour les soins chirurgicaux et anesthésiques.

31. Les budgets pour les soins chirurgicaux devraient être gérés à chaque niveau, c'est-à-dire par le ministère de la Santé, les hôpitaux et les centres de santé. Le budget devrait être dynamique et incrémental.

32. La liste nationale des médicaments et des ressources essentielles devrait inclure l'équipement chirurgical approprié ainsi que les consommables nécessaires.

Déclaration de consensus PASHeF 2023

33. Les pays doivent mettre en œuvre des moyens innovants de financement des soins chirurgicaux.

34. Les pays devraient impliquer le secteur privé pour mobiliser des ressources en vue de renforcer le système chirurgical.

35. Les délégués des pays ont identifié les parties prenantes essentielles dans le processus d'élaboration des plans/politiques de soins de santé chirurgicaux, notamment : les dirigeants au niveau local du gouvernement (ministères de la Santé, ministères des Finances) ; les utilisateurs finaux (groupes de patients et communauté) tels que les groupes de femmes ; le secteur privé et l'industrie ; les sociétés professionnelles ; le monde universitaire ; les ONG ; les forces armées ; l'OMS et d'autres organismes mondiaux ; l'Union africaine ; les organismes de financement tels que la Banque mondiale, le FMI, la BAD ; les organismes régionaux tels que la SADC, la CEA, la WAHO, la CEDEAO, la CEMAC et le COMESA, entre autres.

36. Les ministères de la santé devraient renforcer leurs partenariats avec les organisations internationales, les organismes régionaux et les partenaires de développement.

37. Les partenariats doivent être équitables et transparents.

38. Les parties prenantes doivent être impliquées dès le début du processus d'élaboration du plan/de la politique de soins chirurgicaux.

39. Les délégués des pays ont souligné la valeur des célébrités en tant que défenseurs de la planification/élaboration des politiques chirurgicales.

40. Tous les délégués des pays ont convenu que le plaidoyer au niveau communautaire est essentiel pour promouvoir la prévention des maladies chirurgicales.

Déclaration de consensus PASHeF 2023

41. Le rôle des médias en tant qu'influence positive des plans/politiques de soins de santé chirurgicaux est essentiel pour la mise en œuvre des plans/politiques de soins de santé chirurgicaux en Afrique.

42. L'amélioration des systèmes chirurgicaux devrait être avancée comme une préoccupation de santé publique.

43. Les délégués des pays ont recommandé ce qui suit : faciliter et accélérer l'importation d'équipements ; exemption fiscale pour l'importation de consommables et d'équipements chirurgicaux ; harmonisation régionale des programmes de formation ; système d'approvisionnement régional unifié ; normalisation des lignes directrices et protocoles de prestation des soins ; renforcer la maintenance biomédicale et des infrastructures.

44. Le PASHeF devrait être une plateforme annuelle permettant aux pays de partager leur expérience, de solliciter des échanges et un soutien technique, ainsi que d'engager des partenariats mondiaux.

45. Le PASHeF devrait inviter d'autres partenaires mondiaux lors de ses sessions annuelles.

46. Le PASHeF devrait être accueilli par des pays alternatifs.

47. Chaque pays africain devrait avoir une personne focale représentée au PASHeF.

48. Le PASHeF devrait développer un système de gouvernance inclusif et représentatif de l'ensemble du continent.

49. Tous les délégués des pays ont demandé que ce document de consensus soit officiellement soumis à leurs ministres de la Santé pour une mise en œuvre accélérée.

50. Tous les délégués des pays ont recommandé que les questions de soins chirurgicaux soient inscrites à l'ordre du jour des réunions ministérielles, à la fois lors des réunions de l'Union africaine et des réunions sous-régionales.

Spanish

Declaración de Consenso de PASHeF 2023

1. Los delegados de los países acordaron por unanimidad que los planes/políticas quirúrgicas nacionales son importantes para África.

2. Los delegados de los países acordaron que todos los países africanos deberían completar sus planes/políticas lo antes posible.

3. Los delegados de los países reconocieron que los componentes básicos del plan/política se pueden modificar para abordar las necesidades de cada país.

4. Se hizo hincapié en "no estar casado" con el acrónimo NSOAP (en inglés “National Surgical, Obstetric and Anesthesia Plan”, o “Plano Nacional de Cirugía, Obstétrica y Anestesia”), ya que puede dar lugar a la falta de aceptación por parte de algunas partes interesadas clave.

5. Los delegados de los países acordaron que la implementación de planes/políticas quirúrgicas puede conducir al fortalecimiento de los sistemas nacionales de salud.

6. Los delegados de los países instaron a todos los países a acelerar el progreso hacia la Cobertura Universal de Salud y los ODS para cumplir con los objetivos de 2030 como se especifica en la resolución 68.15 de la AMS.

7. Los países necesitan implementar urgentemente esquemas de seguro de salud que cubran todas las condiciones quirúrgicas.

8. El liderazgo de un "campeón de política/plan quirúrgico" agregaría valor a la planificación/elaboración e implementación de políticas.

9. Sin embargo, todos los delegados de los países advirtieron que los NSOAPs por sí solos no son la solución definitiva a la falta de acceso a la atención quirúrgica en el continente.

Declaración de Consenso de PASHeF 2023

10. El plan/política quirúrgica sirve como base, pero la implementación del plan/política es fundamental.
11. Los gobiernos deben comprometerse a mejorar la infraestructura y el equipo quirúrgico y la estructura de la cadena de suministro en sus países como parte de la implementación del plan/política.

12. Los planes/políticas quirúrgicas recomendadas por los delegados de los países deben diseñarse para servir como componentes integrales del marco de políticas de atención médica existente en los países.

13. Los delegados de los países acordaron que el desarrollo y la implementación de planes/políticas quirúrgicas deben basarse en evidencia y datos locales sólidos (desde el nivel de distrito hasta el nivel nacional).

14. También recomendaron que los países fortalezcan y utilicen sus sistemas nacionales de información de salud y otras fuentes de datos de atención médica para capturar datos quirúrgicos.

15. Los países deben tener herramientas claras de monitoreo y evaluación de la atención quirúrgica, y conjuntos de indicadores.

16. Los delegados de los países reconocieron las siguientes oportunidades en el desarrollo e implementación de planes/políticas quirúrgicas: alianzas regionales para compartir recursos y experiencia; historias de éxito existentes en la planificación/formulación de políticas quirúrgicas en países africanos; una fuerza laboral joven y enérgica en los países africanos.

17. Los delegados de los países identificaron las siguientes barreras para el desarrollo e implementación de planes/políticas quirúrgicas: financiamiento inadecuado para la planificación/elaboración e implementación de políticas; voluntad política reducida y propiedad gubernamental; prioridades en competencia a nivel nacional y local; problemas de recursos humanos (falta de experiencia y motivación que resulta en una fuga de cerebros); la exclusión

sistemática de la atención quirúrgica en las últimas décadas de la atención primaria de salud; escasez de recursos; equipo técnico y apoyo biomédico insuficientes; disparidad entre los presupuestos escritos y la financiación real de la atención quirúrgica.

18. Los delegados de los países acordaron que los pasos propuestos para el desarrollo de la Política Nacional de Atención Médica Quirúrgica son apropiados.

19. Los delegados de los países acordaron que el proceso también debe incluir a la comunidad (usuario final) en la planificación, implementación y liderazgo.

20. Los delegados de los países coincidieron en que las políticas deben centrarse en todo el espectro de la atención quirúrgica (prevención, atención perioperatoria y rehabilitación).

21. Los planes/políticas también deben enfatizar el desarrollo de capacidades, la calidad de la atención, el acceso a la atención y la sostenibilidad.

22. Los delegados de los países propusieron la creación de un sistema panafricano/regional de monitoreo de políticas de atención médica quirúrgica compuesto por estados miembros para dar seguimiento al progreso continental.

23. El seguimiento y la evaluación deben estar alineados con el control de calidad.

24. Los países alentaron la creación de un departamento/dirección de liderazgo de atención quirúrgica dedicado a nivel del Ministerio de Salud con profesionales dedicados y calificados.

25. Los miembros quieren ver planes/políticas quirúrgicas institucionalizados dentro de los sistemas nacionales de salud para permitir la continuidad.

Declaración de Consenso de PASHeF 2023

26. Los países deben incorporar a los ministerios sectoriales interesados en un grupo de trabajo interministerial para el liderazgo conjunto y la supervisión de apoyo.

27. Los delegados de los países recomendaron que este plan/política debería adoptarse al más alto nivel de gobierno (gabinete y parlamento).

28. Los Ministerios de Salud deben crear un comité (grupo de trabajo tecnico) o consejo asesor de expertos para asesorar y monitorear la implementación de los planes/políticas. La estructura de liderazgo también debe reflejarse a nivel de distrito.

29. La OMS debe ser una parte integral de la planificación/elaboración e implementación de políticas, en estrecha colaboración con los Ministerios de Salud.

30. Los delegados de los países recomendaron que los Ministerios de Salud establezcan una línea presupuestaria específica para la atención quirúrgica y de anestesia.

31. Los presupuestos para la atención quirúrgica deben administrarse en todos los niveles: Ministerio de Salud, hospitales, centros de salud. El presupuesto debe ser dinámico e incremental.

32. La lista nacional de recursos y medicamentos esenciales debe incluir el equipo quirúrgico y los consumibles apropiados.

33. Los países deben implementar formas innovadoras de financiación de la atención médica quirúrgica.

34. Los países deben involucrar al sector privado para movilizar recursos para el fortalecimiento del sistema quirúrgico.

Declaración de Consenso de PASHeF 2023

35. Los delegados de los países identificaron a las partes interesadas críticas en el proceso de desarrollo del Plan/Política de Atención Médica Quirúrgica: incluido el liderazgo a nivel del gobierno local (Ministerios de Salud, Ministerios de Finanzas); usuarios finales (grupos de pacientes y la comunidad) como grupos de mujeres; el sector privado y la industria; sociedades profesionales; academia; ONGs; los militares; la OMS y otros organismos mundiales; Unión Africana; agencias de financiación como el Banco Mundial, el FMI, el Banco de Desarrollo Africano; organismos regionales como la SADC, EAC, WAHO, ECOWAS, CEMAC y COMESA entre otros.

36. Los ministerios de salud deben fortalecer sus alianzas con organizaciones internacionales, organismos regionales y socios para el desarrollo.

37. Las asociaciones deben ser equitativas y transparentes.

38. Las partes interesadas deben participar en las primeras etapas del proceso de desarrollo del plan/política de atención médica quirúrgica.

39. Los delegados de los países subrayaron el valor de las celebridades como defensores de la planificación/formulación de políticas quirúrgicas.

40. Todos los delegados de los países acordaron que la promoción a nivel comunitario es esencial para promover la prevención de enfermedades quirúrgicas.

41. El papel de los medios de comunicación como influencia positiva de los Planes/Políticas de Atención Médica Quirúrgica es clave para la implementación de los Planes/Políticas de Atención Médica Quirúrgica Africanos.

42. La mejora de los sistemas quirúrgicos debe promoverse como un problema de salud pública.

Declaración de Consenso de PASHeF 2023

43. Los delegados de los países recomendaron lo siguiente: importación facilitada y acelerada de equipos; importación libre de impuestos de consumibles y equipos quirúrgicos; armonización regional de los currículos de formación; sistema regional unificado de adquisiciones; estandarización de las pautas y protocolos de prestación de atención; fortalecer el mantenimiento biomédico y de infraestructura.

44. PASHeF debe ser una plataforma anual para que los países compartan experiencias, soliciten intercambio técnico y apoyo, e involucren a socios globales.

45. PASHeF debe invitar a otros socios globales a las sesiones anuales.

46. PASHeF debe ser hospedado por países alternos.

47. Cada país africano debe tener una persona focal representada en el PASHeF.

48. PASHeF debe desarrollar un sistema de gobernanza inclusivo que sea representativo de todo el continente.

49. Todos los delegados de los países solicitaron que este documento de consenso se presente formalmente a sus Ministros de Salud para una implementación acelerada.

50. Todos los delegados de los países recomendaron que los temas de la atención médica quirúrgica deberían ser un tema de la agenda en las reuniones ministeriales, tanto en las reuniones de la Unión Africana como en las reuniones subregionales.

Portuguese

Declaração de Consenso PASHeF 2023

1. Os delegados dos países concordaram unanimemente que os planos/políticas cirúrgicas nacionais são importantes para a África.

2. Os delegados dos países concordaram que todos os países africanos devem concluir os seus planos/políticas o mais rapidamente possível.

3. Os delegados dos países reconheceram que os elementos básicos do plano/política podem ser modificados para atender às necessidades de cada país.

4. Houve ênfase em “não ser casado” com o acrônimo NSOAP (em Inglês “National Surgical, Obstetric, and Anesthesia Plan” ou “Plano Nacional de Cirurgia, Obstétrica, e Anestesia”), pois pode resultar na falta de concordância de algumas partes interessadas importantes.

5. Os delegados dos países concordaram que a implementação do plano/política cirúrgica pode liderar ao Fortalecimento dos Sistemas Nacionais de Saúde.

6. Os delegados dos países instaram todos os países a acelerar o progresso em direção à Cobertura Universal de Saúde e aos ODS para atingir as metas de 2030, conforme especificado na resolução 68.15 da WHA.

7. Os países precisam implementar urgentemente esquemas de seguro saúde que cubram todas as condições cirúrgicas.

8. A liderança de um "defensor de plano/ políticas de cirúrgica" agregaria valor ao planejamento/formulação de políticas e implementação.

9. No entanto, todos os delegados dos países advertiram que os NSOAPs sozinhos não são as soluções definitivas para a falta de acesso a cuidados

cirúrgicos no continente.

10. O plano/política cirúrgica serve como base, mas a implementação do plano/política é crítica.

11. Os governos devem se comprometer a melhorar a infraestrutura e equipamentos cirúrgicos e a estrutura da cadeia de suprimentos em seus países como parte da implementação de planos/políticas.

12. Os delegados dos países recomendaram que os planos/políticas cirúrgicas devem ser elaborados para servir como componentes integrais da estrutura de política de saúde existente nos países.

13. Os delegados dos países concordaram que o desenvolvimento e implementação de planos/políticas cirúrgicas devem ser baseados em evidências e dados sólidos locais (desde o nível municipal até o nível nacional).

14. Eles também recomendaram que os países fortalecessem e usassem seus sistemas nacionais de informações de saúde e outras fontes de dados de saúde para coletar dados cirúrgicos.

15. Os países devem ter ferramentas claras de monitoramento e avaliação de cuidados cirúrgicos e conjuntos de indicadores.

16. Os delegados dos países reconheceram as seguintes oportunidades no desenvolvimento e implementação de planos/políticas cirúrgicas: parcerias regionais para compartilhamento de recursos e expertise; histórias de sucesso existentes em planejamento /formulação de políticas cirúrgico em países africanos; uma força de trabalho jovem e enérgica nos países africanos.

Declaração de Consenso PASHeF 2023

17. Os delegados dos países identificaram as seguintes barreiras ao desenvolvimento e implementação de planos/políticas cirúrgicas: financiamento inadequado para planejamento/formulação e implementação de políticas; vontade política e propriedade governamental reduzidas; prioridades concorrentes em nível nacional e local; questões de recursos humanos (falta de experiência e motivação resultando em fuga de cérebros); a exclusão sistemática da atenção cirúrgica nas últimas décadas da atenção básica; escassez de recursos; insuficiente equipamento técnico e apoio biomédico; disparidade entre orçamentos escritos e financiamento real de cuidados cirúrgicos.

18. Os delegados dos países concordaram que as etapas propostas para o desenvolvimento da Política Nacional de Cuidados Cirúrgicos são apropriadas.

19. Os delegados dos países concordaram que o processo também deve incluir a comunidade (usuário final) no planejamento, implementação e liderança.

20. Os delegados dos países concordaram que as políticas devem se concentrar em todo o espectro da assistência cirúrgica (prevenção, assistência perioperatória e reabilitação).

21. Os planos/políticas também devem enfatizar a capacitação, a qualidade dos cuidados, o acesso aos cuidados e a sustentabilidade.

22. Os delegados dos países propuseram a criação de um sistema pan-africano/regional de monitorização da Política de Cuidados de Saúde Cirúrgico composto por estados membros para acompanhar o progresso continental.

23. Monitoramento e avaliação precisam estar alinhados com a Garantia de Qualidade.

Declaração de Consenso PASHeF 2023

24. Os países encorajaram a criação de um departamento/direção de liderança de cuidados cirúrgicos dedicados ao nível do Ministério da Saúde com profissionais dedicados e qualificados.

25. Os membros querem ver os planos/políticas cirúrgicas institucionalizados nos sistemas nacionais de saúde para permitir a continuidade.

26. Os países devem trazer os ministérios dos setores interessados em uma força-tarefa interministerial para liderança conjunta e supervisão de apoio.

27. Os delegados dos países recomendaram que este plano/política seja adotado no nível mais alto do governo (Gabinete e Parlamento).

28. Os Ministérios da Saúde devem criar um comitê (grupo de trabalho técnico) ou conselho consultivo de especialistas para assessorar e monitorar a implementação dos planos/políticas. A estrutura de liderança também deve ser refletida no nível distrital.

29. A OMS deve ser parte integrante do planejamento/formulação e implementação de políticas, em estreita parceria com os Ministérios da Saúde.

30. Os delegados dos países recomendaram que os Ministérios da Saúde estabelecessem uma linha de orçamento específica para cuidados cirúrgicos e anestésicos.

31. Os orçamentos para cuidados cirúrgicos devem ser geridos a todos os níveis – Ministério da Saúde, Hospitais, Centros de Saúde. O orçamento deve ser dinâmico e incremental.

32. A lista nacional de medicamentos e recursos essenciais deve incluir o equipamento cirúrgico e consumíveis apropriados.

Declaração de Consenso PASHeF 2023

33. Os países devem implementar formas inovadoras de financiamento da saúde cirúrgica.

34. Os países devem envolver o setor privado para mobilizar recursos para o fortalecimento do sistema cirúrgico.

35. Os delegados dos países identificaram as partes interessadas críticas no processo de desenvolvimento do Plano/Política de Cuidados de Saúde Cirúrgicos: incluindo a liderança a nível governamental local (Ministérios de Saúde, Ministérios das Finanças); usuários finais (grupos de pacientes e comunidade), como grupos de mulheres; o setor privado e a indústria; sociedades profissionais; academia; ONGs; as forças Armadas; OMS e outros organismos globais; União Africana; agências de financiamento como Banco Mundial, FMI, Banco de Desenvolvimento Africano; organismos regionais como a SADC, EAC, WAHO, ECOWAS, CEMAC e COMESA entre outros.

36. Os Ministérios da Saúde devem fortalecer suas parcerias com organizações internacionais, órgãos regionais e parceiros de desenvolvimento.

37. As parcerias devem ser equitativas e transparentes.

38. As partes interessadas devem ser envolvidas no início do processo de desenvolvimento do Plano/Política de Cuidados Cirúrgicos.

39. Os delegados dos países enfatizaram o valor das celebridades como defensores do planejamento/formulação de políticas cirúrgicas.

40. Todos os delegados dos países concordaram que a defesa a nível comunitário é essencial para promover a prevenção de doenças cirúrgicas.

41. O papel da mídia como um influenciador positivo dos Planos/Políticas de Cuidados de Saúde Cirúrgicos é fundamental para a implementação dos

Declaração de Consenso PASHeF 2023

Planos/Políticas de Cuidados de Saúde Cirúrgicos Africanos.

42. A melhoria dos sistemas cirúrgicos deve ser avançada como uma preocupação de saúde pública.

43. Os delegados dos países recomendaram o seguinte: importação facilitada e rápida de equipamentos; importação isenta de impostos de consumíveis e equipamentos cirúrgicos; harmonização regional dos currículos de formação; sistema de aquisição regional unificado; padronização de diretrizes e protocolos assistenciais; fortalecer a manutenção biomédica e de infraestrutura.

44. O PASHeF deve ser uma plataforma anual para os países compartilharem experiências, solicitarem intercâmbio e suporte técnico e envolverem parceiros globais.

45. O PASHeF deve convidar outros parceiros globais para as sessões anuais.

46. O PASHeF deve ser sediado por países alternados.

47. Cada país africano deve ter uma pessoa focal representada no PASHeF.

48. O PASHeF deve desenvolver um sistema de governança inclusivo que seja representativo de todo o continente.

49. Todos os delegados dos países solicitaram que este documento de consenso fosse formalmente submetido a seus Ministros da Saúde para implementação acelerada.

50. Todos os delegados dos países recomendaram que as questões de cuidados de saúde cirúrgicos fossem um item da agenda das reuniões ministeriais, tanto nas reuniões da União Africana como nas reuniões sub-regionais.
