# Supplementary material for: The Pan-African Surgical Healthcare Forum: An African qualitative consensus propagating continental national surgical healthcare policies and plans
Source: PLOS Glob Public Health. 2024 Nov 12;4(11):e0003635. doi: 10.1371/journal.pgph.0003635 (PMC11556714; doi:10.1371/journal.pgph.0003635)
Supplement: S4 Appendix — (PDF) [file pgph.0003635.s005.pdf]

S4 Appendix. Proceedings and empirical data.

The First Pan-African Surgical Healthcare Forum Report

13-14 July 2023

Kigali, Rwanda

EXECUTIVE SUMMARY

Access to equitable, safe, affordable, timely, and quality surgical healthcare in Africa remains limited. Over 76% of Africans do not live in countries with actionable national surgical healthcare policies. Where such policies or plans exist, there are gaps in dissemination, funding, and implementation. To address these issues, a Pan-African Surgical Healthcare Forum (PASHeF) was initiated to unite policymakers, clinicians, researchers, and advocates. The inaugural forum was a consensus conference of technocrats from African Ministries of Health to discuss challenges, share experiences, and collectively chart the path forward for national surgical healthcare policies and plans. Hosted by the Honorable Minister for Health of Rwanda, Delegates from 53 African countries were invited to a two-day consensus conference in Kigali, Rwanda, in July 2023, focused on national surgical healthcare planning. The forum hosted plenary sessions, working groups, and technocrat networking. A 7-person expert technical working group led discussions. Discussions were sparked by plenary sessions and country experiences, and working groups focused on specific curated context-specific face-validated questions. Documentation involved field notes, audio recordings, and artificial intelligence transcription, and data was coded using a constant comparative method to itemize delegates' observations, declarations, and recommendations, with member checking. A consensus statement was generated through an inclusive decision-making model.

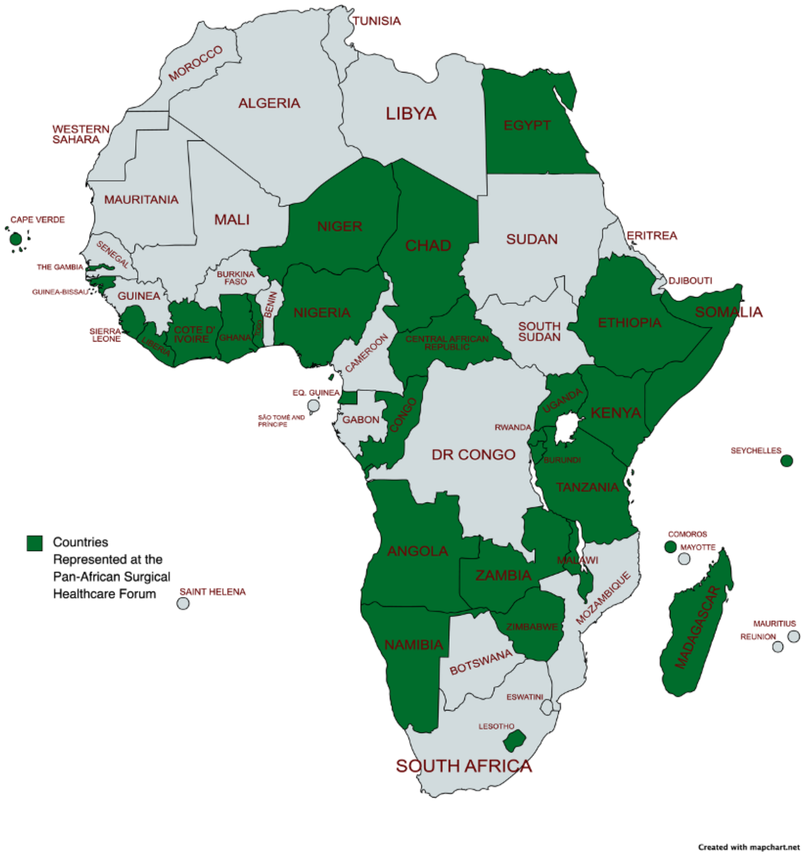

Thirty-two African Ministries of Health (57%) were represented by 42 delegates who drafted and unanimously adopted the PASHeF 2023 Consensus Statement. This was a 50-point consensus addressing country commitment, leadership, financing, stakeholder mobilization, monitoring and evaluation, partnerships, and several other aspects of national surgical healthcare planning in Africa. This consensus is the African roadmap and emphasizes the importance of completing and implementing national surgical health policies, the need for flexibility in policy development, and current opportunities and barriers. It emphasizes that community involvement and sustainability should undergird this planning, in addition to a focus on the entire spectrum of surgical healthcare, including prevention and rehabilitation. Delegates endorsed PASHeF as an annual event with a secretariat and recommended the creation of a Pan-African Surgical Healthcare Policy monitoring system, and that issues of surgical healthcare should be escalated as an agenda item on ministerial meetings, both at the African Union meetings and sub-regional meeting.

African nations have embraced surgical healthcare policy as an imperative on their journey towards Universal Health Coverage. The Pan-African Surgical Healthcare Forum has been endorsed and mandated to provide accountability and serve as a platform for technical exchange and support, and country engagements as well as networking with global partners. It is hoped

that this will galvanize and fast track action to strengthen surgical healthcare across the continent.

Day 01, July 13, 2023

Opening Ceremony

Introduction

Prof. Abebe Bekele, Dean School of Medicine, University of Global Health Equity

Prof. Abebe Bekele, on behalf of the PASHeF technical committee, UGHE, Smile Train, and the Minister of Health (MoH), welcomed everyone to the inauguration of the first Pan-African Surgical Healthcare Forum, PASHeF. He noted that this meeting is a result of a series of conversations between African and African supporters, and that its focus this year is the National Surgical, Obstetric, and Anesthesia Plan (NSOAPs) or the National Surgical Healthcare Plans/Policies.

Prof. Bekele welcomed the 32 African ministries of Health and stated that the forum's objective was to bring together decision-making stakeholders across Africa to discuss challenges, discuss the challenges our continent is facing to improve surgical and anesthesia care, provide platform for sharing experiences, best practices and innovative approaches to strengthen and advance surgical healthcare in Africa. He noted that this year's host is Honorable Minister Dr. Sabin Nsanzimana, Minister of Health of Rwanda, supported by Smile Train and UGHE- Center for Equity in Global Surgery.

Quoting from Ketema Yifru, Ethiopia's representative to the Organization of African Unity and the first Secretary General of the organization he stated, *"We call upon our sister states in Africa to join in the creation under article 52 of the United nations charter of a regional organization of African states, the basic and fundamental task of which will be to furnish the mechanism whereby problems which arise on the continent and our primary interest to the region could, in the first instance, be dealt by Africans, in an African forum, free from outside influence and pressure"*. He emphasized that the discussions completely belonged to the Ministries, and decisions and consensus would be strictly theirs without outside influence.

Message from Mrs. Nkeiruka Obi, Vice President and regional Director, Smile Train

Mrs. Nkeiruka Obi, Vice President and Africa Regional Director of Smile Train, began her speech by highlighting the social and economic consequences of inaccessible surgical care. She noted that disability and catastrophic expenses are common consequences for those who lack access to surgery, and that this can have a devastating impact on communities.

Mrs. NK Obi then spoke about the need to ensure that surgical care is put on the radar in national plans. She emphasized the importance of collaboration between countries, and the use of finance and technology in a way that provides sustainable healthcare. She asked the audience to consider how far we can go together to bridge the gap of 5 billion people who lack access to surgery.

Mrs. Obi then spoke about the importance of focusing on the patients and asking and resolving the tough questions affecting Africa. She emphasized that we need to work together to create sustainable solutions for Africa. She concluded by calling on everyone to stop talking and start acting, and quoted H.E Paul Kagame who stated that, “African stories have been written by others, we need to own our problems and solutions.”

Welcome note: Susannah Schaefer, President Smile Train

Dr. Susannah Schaefer, President of Smile Train, commenced her address by posing a critical question: "Where does Africa stand in terms of surgical healthcare policy?" She proceeded to shed light on the impressive initiatives undertaken by Smile Train across 42 African nations, where essential funding and training are provided to dedicated healthcare professionals. Over the past two decades, Smile Train has methodically honed the skills of thousands of medical experts worldwide, harnessing technology and innovation to tailor solutions to local contexts.

A central theme in Dr. Schaefer's speech was the significance of collaboration. She underscored Smile Train's close partnership with policymakers at the national level, advocating fervently for increased investments in surgical access, obstetrics, anesthesia, and nursing care. She emphasized that the linchpin of this effort was ensuring the affordability of surgical services for all, with policies playing a pivotal role in achieving this crucial affordability. These policies, she stressed, should also prioritize the development of essential soft and technical skills among healthcare professionals.

Partnerships take center stage in Smile Train's approach. Dr. Schaefer highlighted the alliance with KidsOR, a collaboration that facilitates access to operating rooms with the ambitious goal of reaching over 12,000 children worldwide. These partnerships extend support to the training of healthcare professionals, including crucial soft skills like effective intraoperative communication. In a groundbreaking move, Smile Train, in conjunction with KidsOR, is pioneering the use of fully

solar-powered green operating theaters, addressing environmental concerns while simultaneously enhancing patient care in resource-limited settings.

Dr. Schaefer then delved into Smile Train's collaborations with national governments and esteemed institutions such as the College of Surgeons, College of Anesthesia, and College of Medicine in South Africa. These collaborative efforts aim to identify and bridge the gaps that hinder access to surgical care. The utilization of innovative tools, such as the Cleft-E registry for managing clefts and birth defects within communities, serves as a testament to Smile Train's commitment to driving transformative change. Dr. Schaefer passionately encouraged other nations to adopt National Surgical, Obstetric, and Anesthesia Plans (NSOAPs), which, when effectively implemented, have proven to be powerful catalysts for significantly improving patient access to safe, affordable, and timely surgical care.

In conclusion, Dr. Schaefer emphasized that Smile Train's mission extends beyond the realm of cleft care; it constitutes a comprehensive endeavor to enhance surgical healthcare holistically. She underlined the organization's advocacy for critical policy changes, encompassing measures like tax breaks on medical equipment imports and investments in the training and resources of healthcare professionals. Dr. Schaefer stressed the importance of public healthcare provisions, including essential components like nutritional support, all while championing the broad utilization of national health insurance to encompass surgical costs. She concluded with a resounding call to action, affirming that together, we can transform surgical healthcare, one smile at a time.

Opening Remarks: Honorable Minister Dr. Sabin Nsanzimana, Ministry of Health, Rwanda

The Honorable Minister of Health in Rwanda, Dr. Sabin Nsanzimana, began his speech by welcoming delegates to Kigali and thanking them for their dedication and commitment to improving access to surgery. He then highlighted the staggering statistic that approximately 5 billion people worldwide lack access to affordable surgery, and that this shortage has devastating consequences, leading to disabilities and preventable deaths.

The Minister spoke about the importance of training healthcare professionals, procuring equipment, and shaping policies to improve access to surgery. He noted that recent research shows that in Africa, only three countries meet the WHO's recommended physician-to-population ratio of 4:1000. He emphasized that we must strive to meet the minimum requirements and improve healthcare access.

The Minister acknowledged that plans may not always go as expected but encouraged everyone not to be discouraged. He emphasized that small changes could lead to more significant transformations and called upon the attendees to change their mindsets, believing that Africa can achieve a 5:1000 physician-to-population ratio like Europe, or even better.

The Minister then spoke about Rwanda's commitment to change and its goal of transitioning from a 1:1000 physician-to-population ratio to at least 4:1000 in a remarkably short time. He mentioned the introduction of the "4 by 4" program in Rwanda's health sector to quadruple the healthcare workforce, including surgical and anesthesia specialists and midwives.

The Minister proudly announced the forthcoming Center for Excellence in Surgical training in Kigali (IRCAD Africa) as one of 6 centers globally, inviting everyone to be a part of this transformative endeavor. He highlighted the priority of establishing a center for surgery on digestive cancer, equipped with minimally invasive procedures in Kigali. The Honorable Minister encouraged collective efforts to bring about these transformative changes and build a brighter healthcare future for all.

Plenary Sessions

Plenary session 01: What are National Surgical Obstetric and Anesthesia Plans (NSOAPs)?

Speaker: Prof. Emmanuel Ameh, MBBS, FWACS, FACS

The Lancet Commission on Global Surgery identified a significant global gap in access to surgical care, particularly in sub-Saharan Africa and South Asia, where over 95% of the population lack access. This lack of access to surgical healthcare, coupled with the high burden of surgical diseases and complications, has profound implications for individuals, families, and economies. NSOAPs are policy frameworks aimed at addressing this gap and improving surgical healthcare as part of Universal Health Coverage (UHC) and the Sustainable Development Goals (SDGs).

NSOAPs focus on prioritizing surgical healthcare to reduce the burden of surgical diseases and move closer to achieving UHC. However, they have been criticized for their limited focus on non-surgical areas, which are essential for comprehensive healthcare. NSOAPs, on the other hand, aim to promote intersectoral collaboration and integration of surgical healthcare into existing policies and programs. They emphasize the importance of policymaking and recommend prominent placement on Ministry of Health platforms.

NSOAPs offer several benefits, including the streamlining of investments and tracking progress in surgical healthcare. They help identify areas for improvement, reduce costs, strengthen

existing programs, and introduce innovations. However, implementing NSOAPs can face barriers such as funding and finding suitable implementation partners.

To sustain surgical healthcare systems, the first step is to integrate them into existing National Health Plans. Countries can leverage available data sources, such as demographic and health surveys, to plan for surgical healthcare. Incorporating surgical data into existing health information systems and platforms, such as the Department of Health and the District Health Information System (DHIS 2), can facilitate tracking progress and cost savings.

In summary, NSOAPs provide a template for countries to strengthen their surgical healthcare systems tailored to their specific objectives and goals. They are not a rigid prescription but a guide for implementing innovations. By implementing these frameworks, countries can work towards bridging the gap in access to surgical care, improving health outcomes, and reducing the economic burden caused by untreated surgical diseases.

Plenary Session 02: The History of WHA resolution 68:15 and NSOAPs

Speaker: Prof. Emmanuel Makasa

Globally, an estimated five billion people lack access to timely, affordable, and safe surgical care. Most of these people are from the African continent. Surgical diseases can be physically debilitating and can significantly reduce a person's ability to be socioeconomically productive.
The African healthcare system is not equitable, as there are many people in need of surgical care who are neglected. Africa has invested in many things, but surgical care has not been a priority. The resources put into surgical healthcare are very minimal.

Improved surgical care services can help Africa reduce preventable morbidity and mortality. The African healthcare system is organized in a way that district hospitals are the first-level hospitals and receive the most patients. However, during resource allocation, half of the resources are allocated to tertiary-level hospitals, while district hospitals receive only 20% of the resources. This form of resource allocation is not equitable, as resources should be allocated where they are most needed to maintain a healthy population.

Therefore, there is a need for political and technical prioritization to invest in surgical healthcare to achieve universal health coverage (UHC). To achieve UHC, we need to transition from the vertical approaches to health that mainly focus on addressing specific health issues like malaria or HIV/AIDS with separate programs, funding, and infrastructure. Instead, we should adopt a horizontal approach that seeks to integrate and address a wide range of healthcare needs by strengthening healthcare systems to provide universal access to quality health services. This is a

fundamental component of achieving several Sustainable Development Goals (SDGs), including those related to health and well-being. WHA resolution 68.15 holds a pivotal role within the UHC framework, acknowledging the fundamental human right to access safe, affordable, and timely surgical and anesthesia services. It embodies a commitment by member states and calls upon the World Health Organization (WHO) to provide technical support to member states in implementing this resolution.

To put this resolution into action requires translating it into regional frameworks. Each African country should have a National Surgical, Obstetric, and Anesthesia Policy (NSOAP) that can even be contextualized in sub-regions to formulate impactful healthcare policies. This requires the development of technical teams to provide their technical expertise, data-driven insights, and strategic guidance. There is also a need to set up regional collaboration among countries to support each other by creating joint committees through their respective Ministries of Health composed of non-state actors like academic institutions, NGOs, and the private sector.

To develop national surgical policies, many African countries with policies utilized the five key messages and indicators to monitor universal access to safe, affordable, and timely surgical and anesthesia care from the Lancet Commission. They are also based on the four building blocks of the health system which are healthcare delivery and management; workforce, training, and education; economics and finance; and information management. However, a fifth component, which is leadership and governance, was added to assist in stakeholder coordination, accountability, supply chain management, and decentralization of surgical care at the district level.

Key stages of developing a national surgical plan/policy involve first the ownership and leadership of the government, which is the Ministry of Health. Then follows a baseline assessment of the main building blocks to identify potential gaps. Following this, there is stakeholder mobilization to help in the creation of the initial document. Then, there should be monitoring and evaluation of the activities mentioned in surgical plans/policies to assess their implementation, and when they expire, they should be revised. Lastly, the activities should be reported for credibility and stakeholders' motivation.

Even though different countries in Africa are at different stages in the establishment of surgical care policies, the goal is for each African country to develop and implement them. This can be achieved by African countries joining forces to approach surgical care as a team. As His Excellency the President of the Republic of Rwanda, Paul Kagame said, “Let’s write our own story”. Once these policies are in place, they can help to enhance the health of the African people and positively impact their socioeconomic status.

Some of the lessons learned from the review of the Zambian NSOAPs 2017-2021 showed that the implementation of these policies was not guided by a pilot, no implementation took place at the district hospital, and there was no investment for the implementation. Consequently, Zambia struggled to implement the NSOAPs. However, the pilot of the policies prior to implementing them can help generate needed evidence, demonstrate impact, and provide lessons for nationwide scale-up. As a result, it can lead to healthcare systems strengthening, health outcomes improvement, and socioeconomic development of the individual, the community, as well as the nation.

Plenary session 03: NSOAPs: Global status, emphasis on the African continent

Speaker: Prof. Abebe Bekele, MD, FCS, FACS, MAMSE

As of July 2023, the African continent grapples with a stark reality: approximately one billion people, accounting for 76% of its population, lack coverage under active national surgical policies or plans. This deficiency underscores the urgency of NSOAP development across the continent.

In **Senegal**, the Plan Strategique de Developpement de l'Offre De Soins Chirurgicaux (2013-2018) emerged as a pioneering surgical plan, preceding the 2015 Lancet Commission Global Surgery. It is also the first plan to be launched at a national level and fully integrated into the National Health Strategic Plan, although its implementation is to be confirmed.

Ethiopia's first five-year surgical plan, originally titled "Saving Lives Through Safe Surgery" (SaLTS I) and launched independently by the Ethiopian Federal Ministry of Health in 2016, has undergone a renewal. It has been transformed into the "National Surgical Care Strategic Plan" (2021-2025), with substantial effort from the Ministry of Health. Importantly, this updated surgical plan is presently in the process of being implemented.

Zambia, driven by its involvement in the World Health Assembly (WHA) resolution 68.15, introduced the National Surgical, Obstetrics, and Anaesthesia Strategic Plan (NSOASP) for the period 2017-2021. Although currently under revision, Zambia plans to take this initiative to a subnational level, signifying a commitment to surgical policy at all levels of governance.

Tanzania's National Surgical, Obstetrics, and Anaesthesia Plan, 2018-2025, is one of the early plans in Africa and has specifically put emphasis on the cost of national surgical plans. It adapts all six Lancet indicators as a baseline and on numerous other locally derived indicators for implementation.

Rwanda's National Surgical, Obstetrics, and Anaesthesia Plan (2018-2024) emerged through collaborative efforts of the Ministry of Health and the Rwanda Surgical Society. Its implementation is currently under mid-term review.

Madagascar developed its surgical plan, known as the National Plan for the Development of Surgery in Madagascar, for the period 2018-2023 and is actively working towards its implementation.

In **Nigeria**, the National Surgical, Obstetrics, Anaesthesia and Nursing Plan (NSOANP): Strategic Priorities for Surgical Care (StraPS) for the years 2019-2023 is in pilot implementation. Notably, this plan seeks to incorporate the perspective of nursing care and prioritize pediatric surgical care, addressing a crucial aspect of healthcare often overlooked.

Zimbabwe, in a more recent development, launched the National Surgical, Obstetric, and Anaesthesia Strategy (NSOAS) for 2022-2025. Namibia's National Surgical, Obstetric, and Anaesthesia Plan is complete but not yet publicly available. Malawi's Emergency and Critical Care Strategy for 2021-2031 touches on surgical care, though its recognition as a national surgical plan remains unclear.

Beyond Africa, the globalization of NSOAPs is gradually taking shape, with countries in other continents also adopting these plans. Notable examples include Pakistan's pioneering effort in creating the Pakistani National Vision for Surgical Care (2019-2025), which became the first Asian national surgical plan. It also broke new ground by being decentralized, emphasizing the importance of regional adaptation.

In the West Pacific region, strengthening surgical systems has become a regional priority. Member States have endorsed the World Health Organization (WHO) Action Framework for Safe and Affordable Surgery, with varying stages of surgical policy development among countries. Ministers of health have taken up the mantle as champions of this crucial cause.

However, the implementation of surgical policies worldwide is not without its challenges. Limited funding, expertise, political commitment, continuity, and data monitoring and evaluation (M&E) are among the hurdles faced in realizing the full potential of NSOAPs.

In recent years, NSOAPs have gained traction beyond Africa, with at least 11 countries worldwide having implemented surgical policies. Several others, including Malawi, Mozambique, Cape Verde, and Bangladesh, are committed to the development of NSOAPs. This global expansion underscores the growing recognition of the importance of surgical care in comprehensive

healthcare systems, with the hope that these efforts will bridge the gap in access to surgical services and improve healthcare outcomes for populations worldwide.

Plenary Session 04: The Process of NSOAPs Development

Speaker: Dr. Faustin Ntirenganya, MD, MMed, PhD

The development of National Surgical, Obstetric, and Anesthesia Plans (NSOAPs) is a multifaceted process that encompasses several key domains, targets, and procedural steps. Initially, drawing inspiration from the WHO's health system building blocks, the NSOAP framework was structured around six core domains: access to essential medicines, health workforce, service delivery, financing, health information systems, and leadership/governance. However, this framework underwent adaptation by the Lancet Commission, which involved replacing access to essential medicines with a focus on infrastructure and subsequently integrating it into the broader context of service delivery. Notably, the Lancet Commission's initial framework did not include leadership/governance as a core pillar but later recognized its importance and incorporated it.

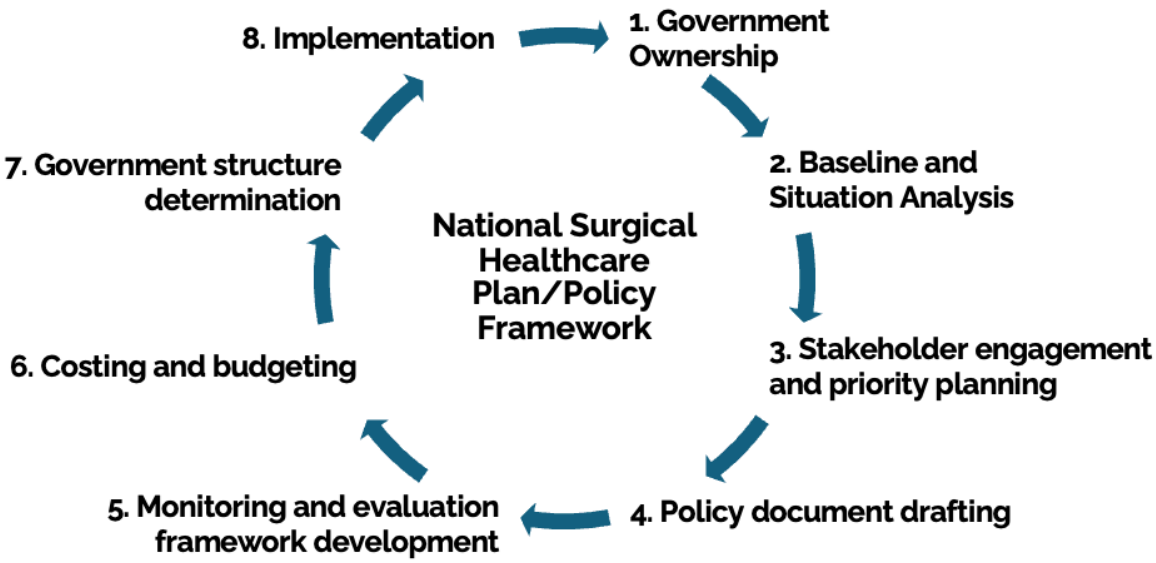

Fig. National Surgical Obstetric and Anesthesia Plan Framework

The NSOAP framework is designed to achieve six pivotal targets, all with the aim of achieving universal coverage of safe, affordable, and timely surgical and anesthesia care by the year 2030. The first target involves ensuring that 80% of the population has access to essential surgical and anesthesia services within a two-hour reach. The second target focuses on maintaining a ratio of

20 surgical, obstetric, and anesthesia providers per 100,000 population. The third target is to establish healthcare facilities capable of performing a minimum of 5,000 procedures per 100,000 population each year in all countries. The fourth target emphasizes the importance of enabling all countries to monitor and track perioperative mortality rates. Target five seeks to protect individuals from the impoverishing expenditure often associated with surgical and anesthesia care. The final target aims to safeguard individuals from incurring catastrophic expenditures related to surgical and anesthesia care.

The NSOAP development process adheres to a well-defined, eight-step theoretical framework designed to guide health policy reforms and enhance access to surgical care while engaging all relevant stakeholders:

1. **Government Ownership:** The process begins with securing support and commitment from the Ministry of Health, ensuring government ownership and involvement.
2. **Baseline and Situational Analysis:** This step involves conducting a comprehensive situational analysis to tailor NSOAPs according to the specific gaps within each country's healthcare system, as well as devising innovative solutions where needed.
3. **Stakeholder Engagement and Priority Setting:** Extensive involvement of experts and stakeholders is crucial at this stage, ensuring a comprehensive and collaborative approach.
4. **Policy Document Drafting:** Technical officers take the lead in drafting the policy documents, drawing from the insights and input gathered from stakeholders.
5. **Monitoring and Evaluation:** Evidence-based decision-making is promoted through the establishment of robust monitoring and evaluation mechanisms, allowing for ongoing assessment of NSOAP implementation and adjustments based on feedback.
6. **Costing and Budgeting:** This step involves gathering stakeholders and experts to secure the necessary resources required for NSOAP implementation and documenting budgetary needs.
7. **Government Structure Determination:** Government commitment is a pivotal step for resource mobilization, policy formulation, and coordination to ensure NSOAP success.
8. **Implementation:** The final step involves the actual implementation of the NSOAP, putting the planned policies and strategies into practice.

Drawing from the experiences of countries that have previously developed NSOAPs, several valuable lessons emerge:

- **Timeline Variability:** The timeline for NSOAP development can significantly vary from one country to another, influenced by local factors and circumstances.

- **Scalability and Regionalization:** Consideration should be given to implementing NSOAPs at subnational levels through pilot programs, allowing for the assessment of their impact before nationwide scale-up.
- **Collaboration:** Encouraging collaboration among academic institutions and professional societies is essential for building research capacity, facilitating data collection, and conducting analysis.
- **Data Collection:** Establishing systematic and sustainable data collection is imperative for informed health sector prioritization, monitoring health financing, and measuring the impact of health reforms.

Developing an NSOAP represents a crucial initial step in strengthening surgical systems and formalizing a nation's commitment to enhancing surgical care. However, the journey from development to successful implementation and scalability is not without its challenges. Addressing these barriers requires engagement and collaboration from a diverse group of national and international stakeholders. Early involvement of ministries of health and finance is critical to advocate for necessary financing amongst competing health system priorities.

Plenary session 05: Experiences of countries on National Healthcare Surgical Plan/policy

Case Study 1: Experience from Ethiopia

Speaker: Dr. Elubabor Buno

Ethiopia, the oldest independent country in Africa and the second most populous after Nigeria, is during a comprehensive development effort aimed at overcoming poverty. As part of this commitment, the Ethiopian government has made significant investments to strengthen its healthcare system, resulting in remarkable progress.

Ethiopia initiated the Health Sector Development Plan (HSDP) in 1997, emphasizing infectious diseases and healthcare infrastructure. Health centers multiplied from 250 to 4,000. Subsequently, Health Sector Transformation Plans (HSTP I & II) prioritized quality improvements in healthcare. However, Ethiopia faces a complex health burden, including communicable diseases, non-communicable diseases, and injuries, necessitating a focus on equity, safety, and quality in healthcare.

Ethiopia's journey began with the Environment Health Matters Initiative (EHMI) in 2006, addressing environmental health, sanitation, water accessibility, and pollution control. In 2007, the blueprint focused on the quality of chronic disease care. In 2010, the blueprint shifted to the Ethiopian Health Reform Initiative Guideline (EHRIG), which focused on hospital reform activities involving emergency, laboratory, and surgical care services. In 2011, the EHRIG shifted to Hospital

Performance Monitoring Initiatives (HPMI) which focused on healthcare providers' training, quality improvement interventions, and healthcare system strengthening. In 2012, came the EHAQ system, a web-based electronic health record that linked University and tertiary hospital providers with primary hospital providers, helping support and mentor primary hospital providers, thus using resources efficiently to build capacity at a lower level.

Then came the Saving Lives Through Safe Surgery Program (SaLTS I&II) in 2016 and 2020 respectively. One of the reasons SaLTS I&II started is the very low surgical volume per year in Ethiopia (289,125 per 100,000 population) compared to the standard set by the Lancet Commission (5000 per 100,000 population). The second reason is a significantly long waiting time for elective surgeries. The third reason is that the surgical workforce number is far below the standards, and most healthcare workers reside in Urban areas. The fourth reason is that surgical site infections are still prevalent and pose major safety issues. The last one is the morbidity and mortality related to the adverse effects of anesthesia. It is in this regard that Ethiopia introduced the first SALT I in 2016 to mitigate these challenges in the field of surgery, to align well with both global and local recommendations, namely WHO & HSTP.

Building upon the progress and achievements of SaLTS I, the SaLTS II program introduced in 2020 continues to serve as the flagship initiative at the national level. It aims to leverage the momentum gained from the previous program to further advance and enhance the state of surgical care in Ethiopia. The guiding principles for the SaLTS program include equity; partnership; innovation; people-centered; professionalism; accountability and transparency.
The SaLTS program in Ethiopia is underpinned by strategic objectives aimed at revolutionizing surgical care. These objectives encompass equitable access to safe surgical and anesthesia care, the improvement of surgical system effectiveness and efficiency, the enhancement of people-centered surgical care, and the reduction of harm stemming from surgical care provision. These objectives align with Ethiopia's commitment to address a myriad of healthcare challenges comprehensively.

The program has set ambitious targets, reflecting its commitment to transformative change in surgical care delivery. These targets include reducing the delay for elective surgery admission from 51 days to 30 days, conducting 2,500 procedures per 100,000 populations by the end of 2025, ensuring 100% access to essential surgical care, reducing anesthesia adverse events by 50%, achieving 100% utilization of the Surgical Safety Checklist, increasing the proportion of health facilities with electricity from 76% to 100%, raising the proportion of health facilities with an improved water supply from 59% to 90%, achieving 100% tracking of surgical care-related deaths, reducing the surgical site infection rate to less than 5%, increasing the percentage of facilities providing basic surgical services from 44% to 80%, reducing the number of clients on the

waiting list for elective surgical service by 50%, reducing the perioperative mortality rate to less than 2%, and increasing the cesarean section rate from 4% to 10%.

To ensure the effective implementation of SaLTS, the Ministry of Health of Ethiopia established the Surgical and Anesthesia Service Desk (SASD). The SASD is a pivotal element of the SaLTS initiative, critical to delivering safe and quality surgical care to all Ethiopians. Comprising three key teams, the SASD covers:

- A national technical working group is providing essential technical expertise and guidance across all facets of surgical care.
- A national surgical care program management team is responsible for the day-to-day execution of the SaLTS initiative, ensuring its integration into healthcare systems.
- A monitoring, evaluation, and learning team, collecting data and meticulously evaluating the SaLTS initiative's impact, thereby facilitating continuous improvement and evidence-based decision-making.

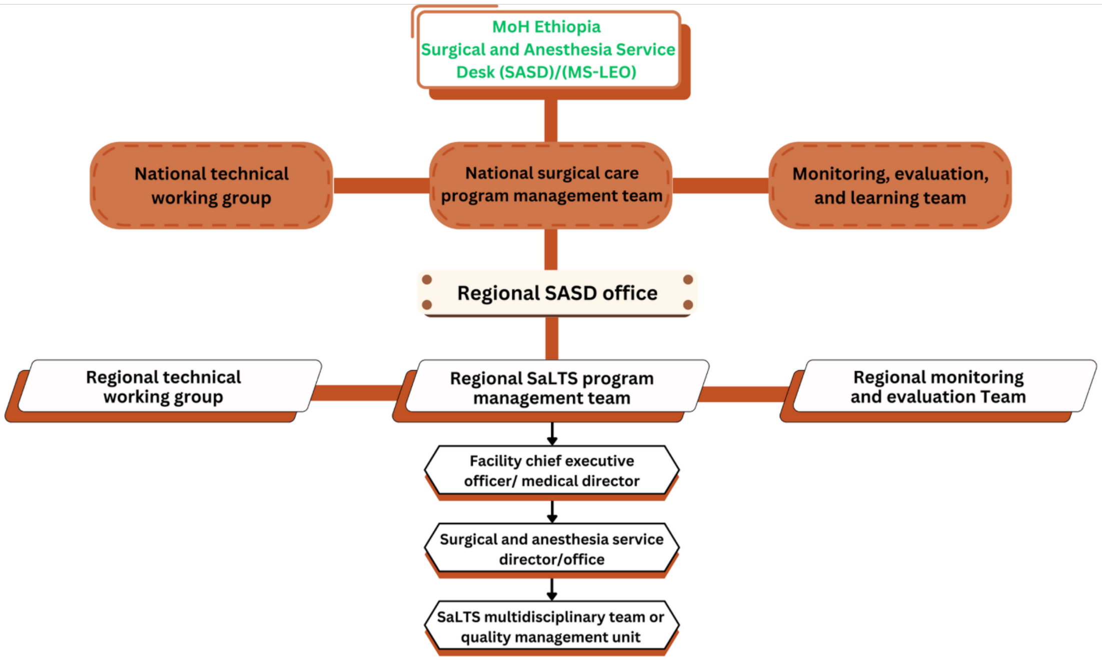

Fig. Organogram showing SALTS governance

SaLTS I, launched in 2016, achieved significant milestones. Annual plans were prepared and budgeted, and a national SaLTS technical working group was established. SaLTS units and teams were formed at the regional and hospital levels, driving progress. Construction of 410 Operating Room (OR) blocks at the Health Center level and renovations of major OR theaters in different regions improved access to safe surgery. Human resource development increased surgical

workforce density, with short-term training for various healthcare professionals promoting the concepts of quality and safety in surgical care.

Seven surgical indicators were integrated into the National monitoring and evaluation tool, bolstering tracking through DHIS2. SaLTS I set a solid foundation for the implementation of SaLTS II, launched in 2020. Key achievements included successful SaLTS I implementation, performance evaluation for the first five years of NSOAP, a community surgical needs assessment, construction of 420 OR blocks in Health Centers, and the development of four Standard Operating protocols, encompassing surgical care, Surgical Nursing Care, Anesthesia care, and Gynecology and Obstetrics care.

Challenges faced during SaLTS implementation include leadership and ownership hurdles, political commitment, restructuring at the Ministry of Health, priority setting, budget allocation, security, and supply-side issues including infrastructure, procurement, and equipment management. Challenges on the demand side comprise a lack of information about healthcare options, direct and indirect costs, socio-cultural, gender, and educational obstacles.

The future direction of the SaLTS program in Ethiopia is anchored in key principles: ownership of the program, further capacity building, incentivizing healthcare professionals, supply provision, infrastructure enhancement, stakeholder engagement, reinforced monitoring and evaluation, improved recording and documentation, and prudent budgeting to ensure the program's sustainability. Ethiopia's journey underscores its commitment to enhancing healthcare quality and access, with the SaLTS programs at the forefront of transforming surgical care and patient safety while addressing multifaceted healthcare challenges.

Case Study 2: Experience from Zambia

Speaker: Dr. Christopher Chanda

Zambia, like many nations, faces significant challenges in ensuring access to essential surgical care for its population. One of the critical challenges Zambia faces is its rapidly growing population. With the current population standing at approximately 20 million, the healthcare system is under immense pressure to provide adequate surgical care. The situation becomes even more complex when considering that a significant portion of the population, estimated at 80%, resides more than two hours away from healthcare facilities capable of providing surgical services. This glaring lack of access to essential surgery raises urgent concerns.

Zambia has shown commitment to global healthcare initiatives, particularly the Sustainable Development Goals (SDGs) that emphasize universal health coverage. WHA Resolution 68.15,

which was sponsored by the government of the Republic of Zambia, recognized surgery and anesthesia as critical components of Universal Health Coverage. However, one stark issue becomes evident: there is no dedicated funding for surgical care within the healthcare budget. Unlike diseases like malaria and HIV/AIDS, which receive substantial funding, surgical care remains financially neglected, leaving a critical gap in resource allocation.

Historical health policies and strategies in Zambia have largely neglected surgical care and anesthesia, focusing instead on maternal and newborn care. This is evident in the WHO Country Cooperation Strategy 2017-2021, the Roadmap for Accelerating Reduction of Maternal, Newborn, and Child Mortality 2013-2016, and the National Strategic Plan 2011-2015. However, there is a growing recognition of the importance of surgical care and anesthesia. A 2018 study by the World Bank found that Zambia has a high burden of surgical conditions, with an estimated 1.5 million people needing surgery each year. The study also found that the country has a shortage of surgical providers, with only 1.1 SAO workforce per 100,000 people.

Regionally, Zambia is part of the Southern African Development Community (SADC), which has demonstrated commitment to healthcare resolutions and strategies. SADC health policies regarding SOA care helped Zambia to commit to resolution WHA 68.1, mobilize funds, formulate NSOAPs, strengthen district-level SOA services (e.g., functionalization of ORs), and develop an SOA information system. Aligning national efforts with regional commitments can strengthen Zambia's position in advancing surgical care.

The process of the Zambian NSOAP planning development started with the situation analysis, strategic focus, monitoring and evaluation, costing and annexes, and the implementation of the plan. After going through the process of NSOAP planning and development, the Zambian government put in place several strategies to implement them. These included:

- Appointing national coordinators in the field of surgery, obstetrics, and anesthesia at the leadership level (national and subnational).
- Appointing the National Safe Surgery Obstetrics and Anesthesia (NSOSA) committee to conduct quarterly meetings to ensure appropriate service delivery and system harmonization.
- Strengthening legal and regulatory frameworks and guidelines, such as those for organ transplants, in vitro fertilization (IVF), and organ donation, to support various surgical procedures.

The importance of an effective NSOAP implementation framework cannot be overstated. Policymakers must ensure that policies and strategies translate into tangible actions. This can be

done by providing adequate resources, ensuring coordination and collaboration between different stakeholders, and monitoring and evaluating the implementation of the plan.
Zambia has rightly recognized the importance of investing in surgical care infrastructure. This commitment is evident in the establishment of the National Heart Hospital for handling referral cases and the upcoming launch of the Women and Newborn Hospital. Additionally, Zambia has taken proactive steps to acquire advanced ICU equipment, demonstrating its dedication to improving healthcare services.

Zambia has taken substantial steps to secure funding for a nationwide health insurance scheme under the purview of the National Health Insurance Management. This initiative, mandated for all citizens, holds the primary objective of alleviating the financial burdens often linked with healthcare expenses. Effective leadership is essential at various levels, including academic institutions and professional societies. These institutions must advocate for the resources and training necessary to advance surgical care. Academic institutions can play a pivotal role in research and rational resource distribution. Scaling up training for anesthetists and other surgical professionals is essential to building surgical capacity. In this regard, Zambia has made significant progress, developing special training programs such as Simulation-based Learning for Anesthesia in Africa, and specialty training programs in surgery, obstetrics and anesthesia to name a few. A focus on non-physician training, supported by doctors, can help to bridge the gap in surgical and anesthesia services. This is because non-physicians can be trained to provide basic surgical and anesthesia care, freeing up doctors to provide more complex care.

District hospitals often serve as the first point of contact for surgical emergencies. Therefore, empowering these hospitals with the necessary skills and resources is paramount. Decentralizing decision-making and resource allocation to district hospitals is vital for expanding surgical care to underserved areas. Political support is vital to securing the necessary resources and commitment to advancing surgical care.

Professional societies can contribute significantly by defining which surgical procedures should be prioritized at district hospitals. This consideration takes into account the skillset available at these facilities, ensuring that life-saving procedures are performed effectively.

Zambia's journey toward ensuring equitable access to essential surgical care is fraught with challenges, but it is also marked by determination and commitment. NSOAPs have emerged as crucial tools in addressing these challenges and building a comprehensive, accessible, and sustainable surgical healthcare system. To achieve this, Zambia must continue to advocate for dedicated funding, integrate surgical care into national healthcare strategies, and invest in training, infrastructure, and workforce development. With leadership, regional collaboration,

and a strong implementation framework, Zambia can bridge the gap in surgical care access and ensure better health outcomes for its growing population.

Case Study 3: Experience from Nigeria

Speaker: Bitrus Oghoghorie Deborah

Nigeria has shown a firm commitment to improving healthcare, including surgical care, to address a myriad of challenges in the country's healthcare landscape. Before the adoption of WHA resolution 68:15, there were substantial issues, including limited access to surgical care at primary and secondary healthcare facilities, inadequate policy attention to surgical care despite the existence of a national healthcare plan, high morbidity and mortality rates associated with surgical procedures, and a staggering rate of medical tourism amounting to 1 billion spent every year, resulting in substantial capital flight. Furthermore, Nigeria witnessed a significant drain of healthcare personnel particularly in the fields of anesthesia and surgery, exacerbating existing shortages, while healthcare leadership, governance, and research were also lacking.

The Nigerian National Surgical Obstetrics, Anesthesia, and Nursing Plan's (NSOANP's) development process was initiated by surgical, obstetrics, anesthesia, and nursing societies and subsequently engaged the Federal Ministry of Health and other stakeholders. An evidence-based approach was adopted, involving a comprehensive baseline assessment of the state of surgical care in Nigeria by particularly focusing on infrastructure, service delivery, human resources, healthcare information, research, financing, governance, and leadership. It was the very first plan to factor in emphasis on care for the high proportion of pediatric patients in sub-Saharan Africa. The plan was then costed, with an estimated budget exceeding $6 trillion. This budget required contributions from various stakeholders, reflecting a collaborative approach.

Notably, Nigeria's NSOANP stood out by recognizing the crucial role of nurses in achieving positive surgical outcomes and prioritizing children's surgery within the plan. Implementation strategies were diverse, including the creation of a dedicated office and the appointment of a desk officer. A committee composed of highly skilled professionals from surgery, nursing, and anesthesia was established. Key entry points were designed to facilitate implementation, and various stakeholders including development partners like Smile Train, EngenderHealth and Physicians' advocacy group, were actively engaged. A successful pilot study was conducted, serving as a blueprint for nationwide scaling.

The implementation of NSOANP in Nigeria has provided valuable lessons. These lessons highlight the importance of involving all stakeholders, government commitment, establishing key points of entry, conducting robust policy dissemination and advocacy efforts, and commencing with

pilot projects on a small scale before scaling up. Nevertheless, challenges were encountered during the NSOANP implementation in Nigeria, including the need to capture the attention of policymakers, secure adequate funding, access technical support, address competing priorities within the Ministry of Health, and convince development partners to provide financial and technical assistance.

The future of the NSOANP in Nigeria holds promising developments aimed at enhancing healthcare delivery. A significant footstep is the development of a comprehensive training manual tailored for primary and secondary-level healthcare providers. This manual encompasses safe adult surgical care, safe C-section procedures, safe children's surgical care (based on the high pediatric population), safe anesthesia safety, and safe perioperative nursing care.

The current NSOANP plan is set to expire by the end of 2023, and plans for its revision are already in motion. This revision process will play a pivotal role in shaping the next phase of NSOANP implementation. The Nigerian NSOANP is set to be incorporated into several critical healthcare frameworks. It will become a crucial component of the upcoming national strategic health development plan and the next child health policy. Additionally, NSOANP data will be seamlessly integrated into the Demographic and Health Survey (DHS) and the District Health Information System 2 (DHIS2), ensuring that it becomes an essential component of Nigeria's broader healthcare landscape.

Case Study 4: Experience from Zimbabwe
Speaker: Shingai Nyaguse

Zimbabwe's National Surgical Strategic and Anesthetic Strategy (NSOAS) was developed in response to a growing demand for surgical interventions, particularly in trauma cases. The government had previously expressed its intention to improve surgical services, but these intentions were not explicitly stated or adequately funded. Additionally, there were significant challenges to decentralizing specialist services due to coordination problems.

The process of developing the NSOAS began in 2018 with an inaugural planning meeting in Harare. Zimbabwe then actively participated in the SADC Ministers' meeting in Namibia in November 2018. A key milestone was reached in 2019 with a stakeholder and partnership mapping workshop at Parirenyatwa Hospital. This laid the foundation for a comprehensive baseline survey, which was conducted in more than 80 hospitals from April to June 2019.

Unfortunately, progress was temporarily stalled by the COVID-19 pandemic. However, the initiative regained full momentum in March 2022. A key writing workshop was held in Kadoma in

July 2022, and the M&E framework and NSOAS document were finalized in August 2022. The NSOAS was officially launched by His Excellency, the Vice President of Zimbabwe and Minister of Health, in September 2022.

Stakeholder engagement was essential to the development of the National Surgical Strategic and Anesthetic Strategy (NSOAS). The process was initially spearheaded by clinicians, who were motivated by their firsthand experiences with patients. However, the development process was slow. These experiences highlighted the urgent need for policy reform. A collaborative strategy that included clinicians, partners, and private sector stakeholders proved to be highly effective. Notably, the engagement of clinicians who would be responsible for implementing the policies was particularly instrumental in the process.

Effective implementation strategies were imperative for the success of the NSOAS. Initially, the landscape and progress were sluggish. However, the introduction of documents to the country's leadership accelerated the process. An important shift was the decision to develop the policy locally rather than relying on external consultants. This approach led to the launch of the policy in 2022, cutting down on costs and leveraging the existing expertise within the Ministry of Health. The plan encompassed a pilot program in four hospitals across two provinces, with plans to integrate surgical policies into existing healthcare structures.

The NSOAP implementation strategy focused on fostering high-level ministry buy-in. Key stakeholders, including clinicians, partners, and the private sector, were actively engaged. Instead of creating a standalone surgical services desk, the approach sought to integrate surgical care into existing healthcare programs and policies.

The National Surgical Strategic and Anesthetic Strategy (NSOAS) is a comprehensive plan to improve surgical care in Zimbabwe. The MOHCC has implemented several effective strategies to ensure the success of NSOAS, including:

- **Fostering high-level ministry buy-in:** The MOHCC has engaged with the Director of Policy and Planning, as well as other senior officials, to ensure that NSOAS has the support of the highest levels of government.
- **Continuous stakeholder engagement:** The MOHCC has engaged with a wide range of stakeholders, such as development partners, private sectors, and clinicians, to ensure that NSOAS is aligned with the needs of the people of Zimbabwe.
- **Maximizing existing programs:** The MOHCC has leveraged existing programs, such as the Smile Train initiatives, Zimbabwe Essential Surgical Training (ZEST), and SAFE-Obs, to save time and resources.

- **Integrating NSOAS into the National Health Strategy:** The MOHCC has integrated NSOAS into the National Health Strategy to ensure that it is not a standalone program, but rather an integral part of the overall health system.
- **Constant monitoring and evaluation:** The MOHCC has implemented a built-in framework for monitoring and evaluating the implementation of NSOAS to ensure that it is effective and efficient.

The MOHCC has also faced some challenges in implementing NSOAS, such as limited funding, lack of human resources, and weak infrastructure. However, the MOHCC is committed to overcoming these challenges and improving surgical care in Zimbabwe.

The NSOAS brought about transformative achievements and impacts in Zimbabwe's healthcare landscape. Previously, a significant portion of healthcare funding was allocated to infectious diseases, leaving a mere 20% for other curative services including surgery. The NSOAS raised awareness of the unmet needs in surgical care, through a baseline study which revealed that only 10% of district hospitals could perform essential surgical procedures. This triggered the change in resource allocation. Surgical camps amplified awareness and facilitated coordination among partners. Data collection also witnessed significant improvements, with trained health information officers at the district level now submitting vital surgical care indicators.

Access to surgical care witnessed notable improvements, with appendectomy camps and plans for tonsillectomy camps on the horizon. Importantly, these initiatives rely on 100% local staff, emphasizing the sustainability of these efforts. Moreover, the introduction of surgical skills training of General Medical Officers (GMOs) at district hospitals under (ZEST), promises enhanced outcomes by allowing low-level surgical staff to perform critical procedures.

Challenges faced included siloed programs within the Ministry of Health, local skills flight, and funding constraints. Valuable lessons for future endeavors highlighted the significance of high-profile champions local champions had passion but poor advocacy skills. Early involvement of the Ministry of Health, and the importance of gaining the buy-in of relevant stakeholders was also a key lesson.

The future of surgical care in Zimbabwe is marked by a commitment to sustainability. M&E efforts will yield reproducible national data, enriching the decision-making process within the Ministry of Health. Plans are underway to enhance domestic funding components, ensuring the long-term viability of surgical care initiatives. Additionally, regional cooperation is set to deepen (e.g., SADC TEWG, WitsSurg, AfroSurg, etc.), creating a more robust network of support and expertise. There

is also a need for a decentralized implementation framework, and the establishment of coordination structures at a national level is a work in progress.

Case Study 5: Experience from Madagascar

Speaker: Rado Razafimahatratra

Madagascar, a vast nation in terms of land area, faces significant challenges in providing surgical care to its population. Presently, the country has only 94 healthcare facilities equipped to deliver surgical services, scattered across 116 districts. This translates to roughly one surgical center for every 33,000 inhabitants, indicating a substantial gap in healthcare accessibility.

The justification for implementing a national surgical development plan in Madagascar aligns with global trends in healthcare reform. Key national and regional documents underscore the importance of strengthening health systems, with a particular emphasis on hospitals. Notable milestones include the adoption of a hospital reform law in 2011, the government's commitment to "health for all at all ages" in 2019, and Madagascar's endorsement of the Dakar Declaration for Horizon 2030, emphasizing surgical development at the district level.

The process of implementing the surgical development plan in Madagascar involved close collaboration and consultation with all relevant stakeholders. The plan aimed to increase access to surgical care for 80% of the population and comprised seven strategic objectives, encompassing geographic access, improved care quality, and financial accessibility. These objectives were translated into a comprehensive plan, with budget allocations at the Ministry of Health.

Madagascar has made remarkable progress in strengthening its healthcare infrastructure and equipment since 2019. The government's investment has led to the construction of 29 district hospitals, each equipped with operating rooms, imaging centers, anesthesia facilities, and laboratories, all soon to be operational. Moreover, Madagascar has taken steps to strengthen blood transfusion centers at the district level, with six already equipped and 24 others awaiting the arrival of essential technical equipment. In a concerted effort to combat maternal mortality, the nation has also established 115 basic health centers specializing in normal childbirth care. Vital support from the Global Fund enabled Madagascar to acquire four oxygen generators and 64-slice scanners, while assistance from BADEA and the World Bank facilitated the purchase of 64 ambulances, with an additional 38 in the delivery pipeline.

The country has also focused on enhancing healthcare human resources, a critical component of surgical development. Although the ideal ratio is four healthcare professionals per 1,000 inhabitants, Madagascar faces resource scarcity and lengthy training periods for surgeons. To address this, a two-year training program for general practitioners as basic surgeons has been

initiated in partnership with Korea, enrolling 20 doctors to serve as basic surgeons for pediatric surgical emergencies (based on the pediatric population's needs), visceral emergencies, and open fractures. The expansion of this program is under discussion in collaboration with Smile Train and the World Health Organization, commencing next year.

In terms of governance and quality of care, Madagascar has developed key documents, including the National Oxygen Therapy Plan which has helped to convince partners to purchase oxygen generators and National Action Plans for infection prevention and control.
Since 2015, Madagascar has promoted the use of operating room checklists at surgical centers which are still operational to date. It has also promoted the use of 5S-Kaizen and Total Quality Management approach, which consists of doing continuous improvement work in terms of service quality. Additionally, the development of a guide on imaging is underway to ensure cost-effective care.

Madagascar has undertaken commendable initiatives to alleviate the financial burden of healthcare on its citizens. In collaboration with UNFPA and Ministère de la Santé Publique et de la Population (MSANP), the country has managed to provide free C/S and delivery surgical kits. It has also conducted campaigns for free cleft palate surgeries with help from Operation Smile. Notably, the MSANP has also initiated campaigns for childhood surgery since 2023, targeting conditions like hernias and cleft palates at the district level. Surgeons from university hospitals extend their services to these districts, offering free care to children afflicted with these conditions.

However, it is essential to highlight that Madagascar faces a significant challenge in terms of healthcare financing. A mere 11% of the population possesses health insurance, with only 9% of this group covered by the Ministry of Economy and Finance, primarily civil servants. This means that approximately 80% of Malagasy citizens must bear their own medical expenses, exacerbating the financial barriers to healthcare access.

While these initiatives represent crucial steps toward improving healthcare access, the ability to measure their impact is impeded by challenges in the development and digitization of information. Madagascar faces difficulties in assessing the true effects of its interventions, hampering the ability to make data-driven decisions and adjustments.

At the national level, several bottlenecks have been identified. Firstly, the COVID-19 pandemic has had a significant impact, disrupting healthcare services for two years. Moreover, there is limited support from technical and financial partners, such as the WHO, USAID, and GAVI, who predominantly focus on community health rather than the hospital level. A complex socio-

cultural aspect also persists, particularly regarding blood transfusions. Despite efforts to establish blood transfusion platforms, the topic remains taboo in many regions, with a dearth of volunteer donors to strengthen hospital blood banks. Additionally, a lack of coordination among professional associations within Madagascar complicates healthcare delivery.

Madagascar faces several primary challenges in advancing surgical care. The foremost challenge is the need for infrastructure and technical platform improvement. Making the National Surgical Development Plan effective and operationalizing the Dakar Agreement are also top priorities. Above all, securing sustainable funding for access to surgical care remains an essential challenge to overcome.

In terms of future prospects, Madagascar is looking to implement and update national surgical plans after comprehensive evaluations. The focus will also be on strengthening and equipping health training institutions, particularly in training and redeploying healthcare personnel at the district level. The digitization of health information and the expansion of telemedicine services are additional avenues being explored. Crucially, Madagascar is actively seeking partners to strengthen the implementation of its surgical development plan, recognizing the importance of collaborative efforts in addressing healthcare challenges.

Case Study 6: Experience from Namibia

Speaker: Francina Marukuavi Ngakuzevi

Namibia's unique context sets the stage for its healthcare challenges and aspirations. With an estimated population of 2.6 million spread across a vast landscape, the nation boasts a low population density of three people per square kilometer. Life expectancy stands at 62.83 years. Namibia is classified as an upper-middle-income country with a Gross National Income per capita of $ 4,729.3, as indicated by the World Bank. However, beneath this surface lies a stark reality, 60.2% of the population lives in poverty or is vulnerable to it, and unemployment affects 33.4% of the workforce.

In alignment with its dedication to providing high-quality healthcare to its citizens, Namibia's healthcare system is divided into two main sectors: the Public Health Sector and the Private Health Sector. The Public Health Sector encompasses an extensive network of 373 healthcare facilities, collectively offering 7,551 hospital beds. This comprehensive system includes one referral hospital, four intermediate facilities, 30 district hospitals, 47 health centers, 291 clinics, and 1,150 outreach points, ensuring widespread access to healthcare services. The Private Health Sector comprises 101 healthcare facilities, providing a total of 1,144 beds, primarily serving the insured population.

The inception of NSOAPs traces its roots back to the Lancet 2015 meeting, which shed light on the escalating burden of surgical diseases globally. This meeting underscored the increasing demand for surgical interventions and identified critical gaps in the delivery of surgical services. To bridge these gaps, recommendations were put forth on how to effectively close them by focusing on the NSOAPs development.

The journey towards formulating NSOAPs commenced in 2018 with the establishment of a task force dedicated to this pivotal initiative. Decision-makers within the Ministry of Health and Social Services (MHSS) played a crucial role in shepherding this endeavor. However, the momentum faced a temporary disruption between 2019 and 2021 due to the COVID-19 pandemic. The NSOAPs regained traction in 2021 with a stakeholder meeting convened to draft the blueprint for surgical and anesthetic strategic planning. In 2022, the task force consolidated these efforts, translating them into a draft ready for the next phase - writing the comprehensive NSOAP document.

Based on the situation analysis data gathered from 29 hospitals, which encompassed 66% from the public sector and 22% from the private sector, it was found that the fundamental infrastructure and tools necessary for delivering emergency Surgical, Obstetric, and Anesthetic (SOA) care were broadly accessible. All hospitals were equipped with essential amenities such as water and oxygen. Remarkably, just one hospital lacked access to electricity, and an impressive 93% of all hospitals possessed operational X-ray and ultrasound machines. However, the availability of internet access was limited to 32% of these facilities, and only one-third of them reported having a consistently available blood bank.

Regarding the bellwether procedures, a significant 83% of healthcare facilities demonstrated competence in conducting cesarean sections, while approximately 55% had the capacity for performing laparotomies, and about 31% were deemed capable of effectively managing open fractures. It is noteworthy that all teaching hospitals highlighted comprehensive surgical care capabilities, offering all bellwether procedures. However, among district hospitals (DHs) 46% of them were equipped to perform laparotomies, and merely 16.7% had the capability to manage open fractures.

The current workforce densities in Namibia fall short of the LCoGS (The Lancet Commission on Global Surgery) target for 2030, which aims for 20 specialist providers per 100,000 population. Achieving this objective necessitates a significant expansion, requiring a fivefold increase in the current density, comprising 1.87 surgeons, 1.47 obstetricians and gynecologists, and 0.68 anesthetists per 100,000 population. Additionally, there is a shortage of other crucial specialist providers essential for delivering surgical, obstetric, and anesthesia (SOA) care, particularly

pathologists and radiologists. Notably, approximately 64% of surgeons, 67% of anesthetists, and 38% of obstetricians and gynecologists practice in Namibia's private sector. Furthermore, access to surgery guidelines is limited, with only 38% of facilities having such access, and approximately 54% reported using the WHO Surgical Safety Checklist. Another interesting finding was that 75% of facilities consistently report Surgical Site Infections (SSI).

The Namibian NSOAP is underpinned by a visionary goal to create a nation of healthy and economically productive individuals. This vision is translated into four strategic objectives:

- **Infrastructure and Equipment:** The plan prioritizes enhancing infrastructural capacity to facilitate efficient and effective delivery of Emergency, Critical, and SOA care.
- **Surgical Workforce:** To ensure that safe SOA services are accessible throughout the nation, the strategy focuses on creating new positions, along with comprehensive training and recruitment of specialized staff across all regions.
- **Service Delivery and Supplies:** Equitable distribution and retention of specialized care providers are central, with an emphasis on optimizing care facilities' performance, timely delivery of high-quality care services, and safeguarding patients from the financial burdens associated with care costs.
- **Information and Management:** A robust data collection system for SOA is a cornerstone, complemented by the development of telemedicine services, reinforcement of data management infrastructure and security, enhancement of audit and research capabilities, and the establishment of telemedicine services for emergency, critical, and SOA care. Improved communication and data dissemination further support these objectives.

Stakeholders in the development of the Namibian National Surgical, Obstetric, and Anesthesia Plan (NSOAP) have played integral roles in shaping its objectives and strategies. Their contributions encompassed the following key functions:

- **Reviewing the Situation Analysis (SA):** Stakeholders actively engaged in scrutinizing the SA, offering their valuable insights to comprehensively understand the current landscape of surgical, obstetric, and anesthesia care in Namibia.
- **Conducting a SWOT Analysis:** Collaboratively, stakeholders conducted a SWOT analysis, identifying the strengths, weaknesses, opportunities, and threats within the healthcare system. This analysis provided critical input for strategic planning.

- **Drafting the NSOAP:** Stakeholders played a pivotal role in the drafting of the NSOAP. Drawing from their diverse backgrounds and expertise, they ensured that the plan was holistic, well-informed, and aligned with the specific needs of Namibia.

A total of 44 stakeholders participated in this endeavor, representing various facets of the healthcare sector, including the Ministry of Health and Social Services (MHSS) with professionals ranging from medical officers, nurses, surgeons, obstetricians/gynecologists, medical technicians, HR practitioners, primary healthcare program officers, and the quality assurance team. Additionally, key training institutions such as UNAM, IUM, and NHTC, along with the World Health Organization (WHO) and experienced consultants specializing in NSOAP development like SADC-Wits Regional Collaboration Centre on surgical healthcare (WitSSurg) and Global Surgery Fellows from esteemed institutions like Harvard Medical School's Program in Global Surgery for Social Change (PGSSC) and the University of the Witwatersrand's WitSSurg Centre, provided essential expertise.

Reflecting on this collaborative effort, several lessons were learned. The importance of political will emerged as a driving force behind any healthcare initiative within the country. Stakeholder engagement was recognized as a critical component throughout the development and planning phases of the NSOAP, ensuring that a wide spectrum of perspectives was considered. Moreover, there was a clear recognition of the need for technical support and benchmarking from experienced entities to guide the successful implementation of the plan.

Looking forward, the next steps for the NSOAP involve estimating the costs, with valuable support from the WHO. The plan is set to be officially launched at the end of July 2023, marking a significant milestone. The initial phase will involve implementing a pilot program to test and refine the strategies outlined in the NSOAP.

In conclusion, the Namibian NSOAP stands as a comprehensive and forward-looking framework designed to address the intricate challenges presented by emergency and essential surgical, obstetric, and anesthesia conditions in the nation. Operating within six major health system domains, which encompass surgical infrastructure and equipment, surgical health service delivery and supplies, surgical health workforce, surgical health information management and quality improvement, surgical health financing, and surgical health system governance, the NSOAP is poised to enhance healthcare delivery in these vital areas. Its development and future implementation highlight Namibia's commitment to providing accessible, high-quality care for its citizens.

Case Study 7: Experience from Rwanda

Speaker: Dr. Parfait Uwaliraye

In line with The Lancet Commission's indicators for global surgery, Rwanda established its baseline in 2017, serving as a reference point for measuring progress. These indicators encompass surgical volume, perioperative mortality tracking rates, access to timely essential surgery, specialist surgical workforce, surgical safety, and financial protection against surgical expenses. This baseline assessment initiated Rwanda's commitment to improving surgical, obstetric, and anesthesia care within the country.

The World Health Assembly's resolution further guided Rwanda's path toward developing and implementing the NSOAP. This resolution called for strategic interventions to enhance surgical and anesthesia care while aiming to ensure access to quality surgical care without causing financial hardship for citizens.

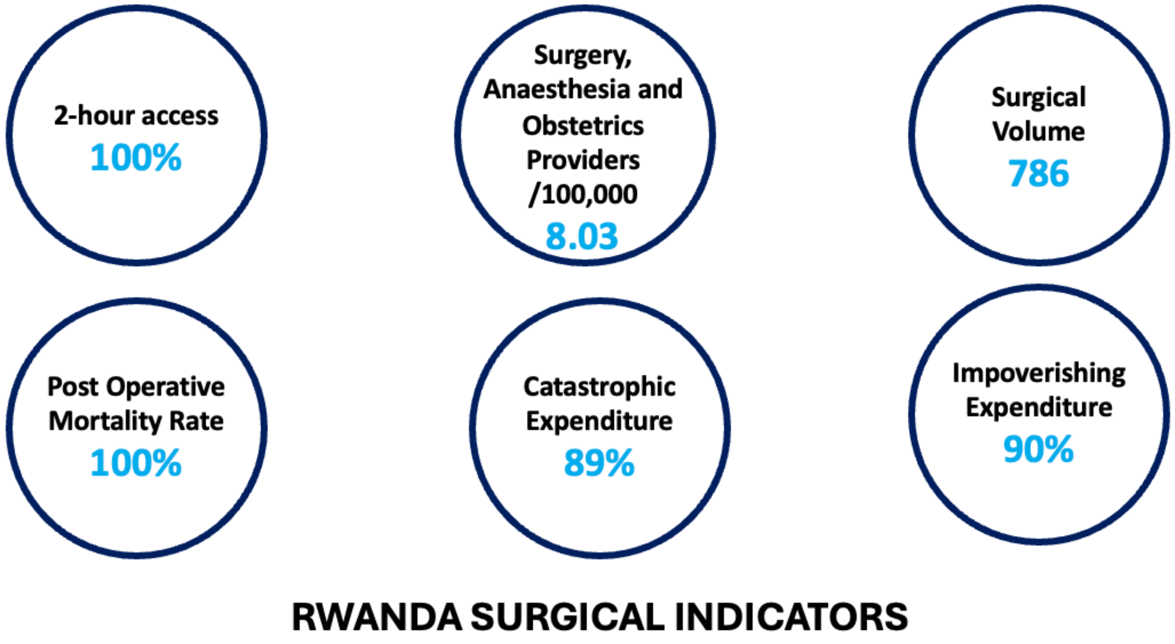

Fig. Rwanda's surgical indicators in 2017

The Rwanda NSOAP's strategic interventions align with the World Health Organization's building blocks and outlines a comprehensive strategy to enhance healthcare services, focusing on various key strategic interventions:

- **Infrastructure:** Rwanda recognizes the importance of essential resources such as water and oxygen and is optimizing equipment availability in district operating rooms to ensure efficient surgical care delivery.

- **Service delivery:** The NSOAP aims to achieve equitable and standardized care through the redistribution of SAO professionals to underserved areas, enhancement of the referral system, and establishment of standardized protocols for surgical care in referral and provincial hospitals.
- **Workforce development:** Rwanda is addressing the shortage of SAO professionals by expanding specialty and subspecialty training programs, reinforcing CME for SAO providers, and strengthening intern training programs to include hands-on experience.
- **Information management:** A robust information system will be established to facilitate data-driven decision-making and ensure efficient resource allocation. Research initiatives will also be promoted to enhance SAO care quality.
- **Finance:** The NSOAP aims to reduce impoverishment and catastrophic expenditure rates for uninsured patients seeking surgical services, ensuring that financial constraints do not impede access to necessary care.
- **Governance:** The Ministry of Health (MoH) will play a pivotal role in overseeing NSOAP implementation, ensuring accountability, and coordinating efforts across stakeholders.

Rwanda has made significant strides in its NSOAP journey. Surgical volumes have increased, reflecting improved access to surgical care. However, challenges remain, including the underreporting of data from healthcare facilities. Efforts are underway to enhance data reporting and analysis, working closely with professional societies to improve accuracy.

Indicators	FY 2016-2017	FY 2017-2018	FY 2018-2019	FY 2019-2020	FY 2020-2021	FY 2021-2022
Surgical volume per 100,000 population	1,005	1,098	1,255	1,128	1,281	1,325
Perioperative mortality tracking rate	NA	76.2	100	100	100	100
Perioperative mortality rate	0,6	0,6	0,5	0,6	0,5	0.43
SAO provider density per 100,000 population	NA	NA	11	12	12	12

Rwanda's experience also highlights the importance of increasing the number of surgical care providers. To address this, the government has adopted strategies such as credentialing and privileging health professionals, allowing them to perform essential surgical procedures (bellwether procedures) confidently.

Rwanda's NSOAP continues to evolve, with plans to develop an implementation strategy based on lessons learned from the Mid-Term Review (MTR). The government aims to upgrade healthcare facilities to teaching hospital standards, enhancing both equipment and workforce. Additionally, there is a focus on increasing the number of trainees in surgical and anesthesia care.

Day 01: Breakout Sessions

Table 1: Day 01 Breakout Session Groups

	Breakout Group Focus	Breakout Discussant Countries
1	**Are National Surgical Policies/Plans relevant to Africa?**	Angola, Burundi, Cabo Verde, Central African Republic, Chad, Comoros, Congo, Côte d'Ivoire, Egypt, Equatorial Guinea, Ethiopia, Gambia, Ghana, Guinea-Bissau, Kenya, Lesotho, Liberia, Madagascar, Malawi, Namibia, Niger, Nigeria, Rwanda, Seychelles, Sierra Leone, Somalia, Somaliland, Tanzania, Togo, Uganda, Zambia, Zimbabwe
2	**Are National Surgical Policies/Plans important to your country?**	Angola, Egypt, Ethiopia, Ghana, Gambia, Guinea Bissau, Kenya, Lesotho, Malawi, Namibia, Nigeria, Sierra Leone, Somalia, South Sudan, Tanzania, Uganda, Zimbabwe
3	**What are the perceived advantages**	Angola, Egypt, Ethiopia, Ghana, Gambia, Guinea Bissau, Kenya, Lesotho, Malawi, Namibia, Nigeria, Sierra Leone, Somalia, South Sudan, Tanzania, Uganda, Zimbabwe
4	**What are the perceived disadvantages**	Angola, Egypt, Ethiopia, Ghana, Gambia, Guinea Bissau, Kenya, Lesotho, Malawi, Namibia, Nigeria, Sierra Leone, Somalia, South Sudan, Tanzania, Uganda, Zimbabwe
5	**What are the main opportunities African countries have in the development of National Surgical Policies/Plans**	Angola, Egypt, Ethiopia, Ghana, Gambia, Guinea Bissau, Kenya, Lesotho, Malawi, Namibia, Nigeria, Sierra Leone, Somalia, South Sudan, Tanzania, Uganda, Zimbabwe
6	**What are the main potential barriers to the development of National Surgical Policies/Plans**	Angola, Burkina Faso, Burundi, Cabo Verde, Central African Republic, Chad, Comoros, Congo, Cote D'Ivoire, Equatorial

		Guinea, Kenya, Liberia, Madagascar, Mauritania, Niger, Rwanda, Somaliland, Sudan, Seychelles, Togo, Zambia
7	**What are the main potential barriers to the implementation of National Surgical Policies/Plans**	Angola, Burkina Faso, Burundi, Cabo Verde, Central African Republic, Chad, Comoros, Congo, Cote D'Ivoire, Equatorial Guinea, Kenya, Liberia, Madagascar, Mauritania, Niger, Rwanda, Somaliland, Sudan, Seychelles, Togo, Zambia
8	**Are the proposed steps of National Surgical Plan development appropriate?**	Angola, Burkina Faso, Burundi, Cabo Verde, Central African Republic, Chad, Comoros, Congo, Cote D'Ivoire, Equatorial Guinea, Kenya, Liberia, Madagascar, Mauritania, Niger, Rwanda, Somaliland, Sudan, Seychelles, Togo, Zambia
9	**Who are the major stakeholders in the process of National Surgical Policy /Plan development, and how can they be brought onboard?**	Angola, Burkina Faso, Burundi, Cabo Verde, Central African Republic, Chad, Comoros, Congo, Cote D'Ivoire, Equatorial Guinea, Kenya, Liberia, Madagascar, Mauritania, Niger, Rwanda, Somaliland, Sudan, Seychelles, Togo, Zambia
10	**How shall African modify the steps?**	Angola, Burkina Faso, Burundi, Cabo Verde, Central African Republic, Chad, Comoros, Congo, Cote D'Ivoire, Equatorial Guinea, Kenya, Liberia, Madagascar, Mauritania, Niger, Rwanda, Somaliland, Sudan, Seychelles, Togo, Zambia

Discussion and Report

1. Are NSOAPs relevant to Africa?

Delegates agreed that NSOAPs are relevant to Africa, however it was mentioned the imperative need to strengthen the overall health system to effectively develop and implement NSOAPs. While NSOAPs are pivotal as a foundation element, their true impact hinges on effective implementation. The delayed occurrence of bilateral collaboration between neighboring countries is also a concern. Aligning existing guidelines and policies to steer NSOAPs is crucial, as is designing a responsible body for their development, implementation, and ongoing monitoring. This necessitates a thorough examination of financing structures and a comparative analysis of

NSOAP's budget within the context of the broader healthcare budgets. Furthermore, promoting horizontal funding and investments is essential, with a primary focus should be on extending healthcare coverage to underserved rural communities.

2. Are NSOAPs important to your country?

NSOAPs were labeled as fundamentally important by delegates to tackle the growing burden of surgical disease in their respective countries. The majority of the delegates stated that efforts with NSOAP development and implementation are often delayed, underscoring the pressing need to address surgical healthcare needs. NSOAPs are perceived as a catalyst to bring innovative and advanced approaches that can lead to enhanced surgical services and achieve equitable distribution of surgical workforce and infrastructure. Moreover, it was noted the need to make NSOAPs more holistic and integrated with other local policies and strengthened through collaboration between neighboring countries. Delegates also mentioned that NSOAPs should transcend national boundaries to evolve into a global movement, which can make a significant contribution to achieving Sustainable Development Goals.

3. What are the perceived advantages?

According to delegates, NSOAP is crucial for strengthening the overall healthcare system, making it resilient and responsive to population health needs. Numerous specific advantages were discussed, including serving as a platform to increase the visibility of surgical needs and help establish a strong relationship between stakeholders. This will serve as a valuable tool for securing the necessary funds for surgical care and to mobilize resources effectively. The integration of Surgery, Obstetrics, and Anesthesia will further help optimize resources and emphasized the importance of including perioperative management in the plan. NSOAPs will contribute to enhancing access to timely quality surgical services while reducing mortality, disabilities, and poverty. Additionally, it will provide a framework for effective monitoring and evaluation of surgical services, ensuring continuous progress.

4. What are the perceived disadvantages?

There were several noteworthy points raised regarding the potential disadvantages of NSOAPs. These included the need for increased budget allocation to support the implementation of NSOAPs that would compete with the existing healthcare budget and highlighted the potential issue of duplication of policies. Delegates recommended a country-specific approach to determine the most effective placement or integration of NSOAPs to avoid conflict with existing plans. Another point of discussion was the balance between treatment and prevention, with some suggesting emphasis is needed on prevention in NSOAPs. Additionally, there were concerns about shifting policy prioritization and implementation with changes in leadership. Shortage of

specialized care, insufficient partnership, and lack of research and data were identified as challenges to be addressed.

5. **What are the main opportunities African countries have in the development of National Surgical Policies/Plans?**

Delegates at the conference underscored a range of opportunities for Africa in the development of national surgical plans and policies. It can garner a positive impression, that can lead to endorsements by the World Health Organization (WHO) with a subsequent funding for implementation. There is potential for integration with other programs such as Maternal and Child Health (MCH) programs, resulting in a positive impact on MCH outcomes that can lead to additional funding. Increased awareness regarding the critical importance of surgical care and potential consequences arising from the lack of quality surgical care, can lead to greater support and investment from government bodies, ministers of health, and partner organizations. The National Surgical policies/plans offer networking opportunities for learning from countries who have already implemented them, fostering collaboration and the sharing of resources and human resources. Additionally, it can help boost a country's economy sector.

Delegates noted the existing building blocks for national surgical plans and policies can serve as the foundation that can be modified to suit the specific needs and context of each African country. These plans/policies can help to optimize resources by combining surgery, obstetrics, and anesthesia, and Integration in strategic direction to prioritize communities in need such as rural areas. The presence of young and energetic surgeons within ministries of health was mentioned as a unique advantage, fostering the development and ownership of policies and plans within the ministry. It can also provide a platform for research and training for the workforce. Another significant avenue identified was the need for public sensitization particularly via grass root advocacy to position surgery as a public health concern on the African continent. It was further emphasized that there is a necessity to redirect attention from treatment to prevention, as preventing conditions is deemed more feasible than undertaking surgical interventions.

6 & 7. What are the main potential barriers to the development and implementation of National Surgical Policies/Plans?

Delegates outlined the following obstacles to the development and implementation of surgical plans/policies: insufficient funding for planning and policy execution; diminished political commitment and government ownership; conflicting priorities at both national and local levels; the historical exclusion of surgical care from primary healthcare in recent decades; human

resource challenges, encompassing shortages in expertise, training, and motivation, along with retention issues due to brain drain; inadequate technical equipment and biomedical support; discrepancies between allocated budgets and actual funding for surgical care. Additionally, they highlighted a lack of capacity building and the integration of National surgical healthcare policies/plans into standard practice.

7. Are the proposed steps of National Healthcare Surgical Plan/ Policy development appropriate?

Country delegates agreed the proposed steps of National Surgical Healthcare Plan/ Policy development are appropriate. However, they emphasized the need for contextualization based on the need and priority of the specific country.

8. Who are the major stake holders in the process of National Surgical Policy /Plan development, and how can they be brought onboard?

Country delegates identified critical stakeholders in the process of Surgical Healthcare Plan and Policy development: including local governmental level leadership (MoH, Ministries of Finance); end users (patient groups and the community) such as women groups; the private sector and industry; professional societies; academia; non-governmental organizations; the military; the World Health Organization and other global bodies; African Union; funding agencies such as World Bank, International Monetary Fund, the African Development Bank; regional bodies such as the South African Development Community, the East African Community, the West African Health Organization, the Economic Community of West African States, Communauté Économique et Monétaire de L'afrique Centrale and The Common Market for Eastern and Southern Africa, among others. Ministries of Health should strengthen their partnerships with international organizations, regional bodies, and development partners. Stakeholders should be engaged early in the process of Surgical Healthcare Plan and Policy development. However, these partnerships should be equitable and transparent.

9. How shall African modify the steps?

Country delegates agreed that the proposed steps of National Surgical Healthcare Policy development in the United Nations Institute for Training and Research National Surgical planning manual are appropriate. However, they noted that not enough emphasis has been placed on the role of the community within the framework of the roadmap to implementation. Country delegates agreed that the process should also include the community (end user) in planning, implementation, and leadership. Delegates insisted that beyond a focus on management of

surgical conditions and provision of surgical healthcare for established surgically treatable disease, national surgical healthcare policies should incorporate community-based prevention of surgical diseases and rehabilitation. All country delegates agreed that community level advocacy is essential to promote prevention of surgical diseases.

In addition, for African countries, policies should emphasize capacity building, quality of care, access to care and sustainability. The framework should include an emphasis on the ethos of sustainability in various dimensions. Sustainability in this sense refers to "a state in which disadvantaged communities or developing countries can address health challenges and provide quality, equitable healthcare to their populations with limited reliance on external support". This includes the ability of the national surgical healthcare plan to continue to function effectively, for the foreseeable future, with high utilization, integrated into available health care services, with strong community ownership using resources mobilized by the local community and government. Environmental sustainability should also be part of the sustainability ethos undergirding national surgical healthcare planning.

Day 01: Summary and Key takeaways

1. Participants unanimously agree that national surgical plans are relevant to Africa. 95% of people who lack surgical care are based in Africa, and the continent has the largest burden of trauma, increasing incidence of NCDs, associated with high morbidity and mortality. Surgical volume and the number of practitioners is also very low.

2. Participants stressed that designing such plans alone is not the solution - surgical plans serve as the foundation, but implementation of the plan is critical which is the bane of any health policy.

3. Ministry representatives agree that apart from being relevant to the continent as a whole, national surgical plans are important to their specific countries as the plans provide a template for strategic direction, integration with other health sectors, strengthens the overall health system and creates a system of accountability.

4. NSOAPs implementation will lead to entire Health Systems Strengthening as provision of Safe Surgical healthcare requires that all aspects of the health system functioning at optimal capacity (Laboratory services, Imaging, ICU, referrals, Blood Transfusions, critical care).

5. Countries need to accelerate progress towards UHC and the SDGs considering we have only 7 more years to reach the 2030 deadline. Countries need to implement schemes for insurance and universal health coverage.

6. National surgical policies/plans have various **advantages** including:

- Relationship building between stakeholders
- Reducing morbidity and mortality
- Improve several other areas of healthcare including maternal and child health, NCDS, infectious disease and pandemic mitigation
- Increase visibility of surgical care by unearthing of unmet need
- Poverty reduction
- Improve quality in healthcare delivery
- Reduce waiting times,
- Improve advocacy for surgical care

7. The perceived **challenges** to national surgical planning include:
    - It may lead to more focus on treatment rather than prevention of surgical diseases
    - The need for budget and significant expansion of infrastructure
    - Lack of research and data that is critical to the designing of the plans
    - Recurrent change at ministry level leadership that can lead to instability of the policies
    - Shortage of specialized care practitioners
    - Insufficient global and regional partnerships
    - National surgical policy may be perceived as in competition with policies that are already in place.
    - The risk of National Surgical Healthcare Policies becoming another siloed program.

8. Countries need to use their DHIS and other healthcare data sources in planning and implementing their surgical plans.

9. Participants recognize that duplication might be an issue (with maternal and child health plans) and recommend the need to find ways on how to best place or integrate surgical plans within existing policy frameworks

10. Countries recognized that the opportunities African countries have in the development of Surgical Healthcare Policy include
    - Sharing of resources - Countries who have successfully developed and implemented their Policy are assets of Africa as they can guide others and share their mistakes and successes
    - Regional partnerships
    - Existing success stories in surgical planning in some countries
    - Communities - Public sensitization, and grassroots advocacy
    - Research and learning
    - A young and energetic workforce
    - Resource optimization by combining focus on Surgery, Obstetrics and Anesthesia care.
    - Having building blocks that can be modified based on country needs

11. The **main barriers** to the development and implementation of National Surgical Healthcare Policies include:
    - Inadequate financing for planning,
    - Reduced political will and governmental ownership,
    - Competing priorities,
    - Lack of expertise in planning and implementation
    - Human resource issues including lack of training and motivation which results in brain drain.
    - The systematic exclusion of surgical care in the last few decades from Primary Health Care
    - Shortage of resources
    - insufficient technical equipment and biomedical support,
    - Disparity between written budgets and actual financing of surgical care

12. The proposed steps of National Surgical Healthcare Policy development are deemed appropriate, however, there is the need for contextualization:
    - Participants stressed the need to include the community in planning, implementation and leadership
    - African Surgical Healthcare Policies should incorporate a focus on the entire spectrum of surgical care including
        - **i.** prevention,
        - **ii.** perioperative care
        - **iii.** rehabilitation.
    - Plans should emphasize quality of care
    - Plans should emphasize accessibility to care
    - Plans should emphasize on Sustainability

13. The role of the media as a positive influencer of Surgical Healthcare Policies is key for the implementation of African Surgical Healthcare Policies.

14. The major stakeholders in the process of Surgical Healthcare Policy development were identified as:
    - Local Governmental level leadership (MoH, Ministries of Finance)
    - End users (patient groups and the community) such as women groups
    - The Private sector and industry
    - Professional societies
    - Academia
    - NGOs
    - The Military
    - WHO and other global bodies
    - African Union,
    - Funding agencies such as World Bank
    - Regional bodies such as the SADC, EAC, WAHU, ECOWAS and COMESA

15. Partners should be engaged early in the process of Surgical Healthcare Policy development

16. Participants put forward the suggestion that a Pan-African Surgical Healthcare Policy monitoring committee composed of member states should be set up and commissioned to review and follow up the progress of countries in Surgical Healthcare Policy development and implementation.

17. Countries strongly encouraged the creation of a dedicated surgical care leadership department or Surgical Healthcare Policy desk office at the level of ministries of health with dedicated desk officers and professionals.

18. Members want to see surgical policy carried forward from one government in power to the next, independent of political interests, as this has stalled national surgical planning in many African countries.

19. There was emphasis on "not being married" to the acronym NSOAP, as it may result in lack of buy-in from some key stakeholders.

20. Other recommendations and their domains are below

Governance

- Enforce MOH to prioritize and the leadership in surgical care planning and implementation
- Integrate NSOAPs to the existing national health plan
- Overall improving the health system will help in implementation of NSOAPs
- Political/government commitment
- Decentralization (involve local/district level)
- Establish surgical care leadership division or desk at the MoH level

Finance and resources

- Comparative analysis of NSOAP budget compared to overall health budget
- Horizontal funding and investment
- Holding WHO accountable to prioritize funding for surgical care
- Infrastructure and equipment

Monitoring and Evaluation

- Aligning guidelines for NSOAPs implementation
- Responsible body for Implementation and follow-up of NSOAPs (Pan African monitoring and evaluation committee)

Partnerships/stakeholders

- Collaboration between different stakeholders (local, government/army. private, civil society, regional (AU), global (WHO, World bank)
- Patients as a stakeholder (insurance, emergency surgery phone line)
- Involve partners from the initiation phase of NSOAPs planning and development

Advocacy and community engagement

- Community awareness to increase prevention
- Utilization of media: increase awareness/sensitize communities and show government and leaders how NSOAPs are implemented with ownership (improve political will)

Training and service

- Include perioperative management in NSOAPs
- Training of mid-lower personnel/non-physicians

Day 02, 14 July, 2023

Plenary Session 05: Experiences of countries on National Healthcare Surgical Plan/policy

Case Study 8: Experience of Angola

The presentation on Angola highlighted the country's efforts in healthcare development and improving surgical services. The National Development Plan (PND) for 2018-2022 focuses on addressing infectious diseases as well as emergency and elective surgeries. The plan includes the equipping of hospitals and training of healthcare professionals, with two large public tenders admitting 33,093 new professionals in 2018 and 2019. The goal is to increase the number of doctors, nurses, and midwives significantly, with plans to expand medical schools and reduce the doctor-patient ratio. Actions such as the creation of the Institute of Health Specialization and curriculum development have been promoted to enhance staff training. Specifically addressing obstetric surgeries, Angola is training anesthesiologists, instrumentalists, and traditional midwives to address the doctor-patient ratio in this area. Beyond human resource development, improving infrastructure and hospital equipment is also a priority. This includes the construction of hospitals and health centers and the introduction of technologically advanced equipment, such as robotic surgery, which aims to reduce the need for people to travel abroad for specialized procedures. The government has also invested in structural or systemic improvements. For instance, identifying trauma as the second leading cause of mortality after malaria, the government established a dedicated body to address this issue. By 2050, Angola aims to reduce maternal mortality, perform surgeries at all levels of care, increase healthcare expenditure, and improve the doctor-to-population ratio. These goals reflect the country's commitment to improving healthcare services and expanding access to surgical care.

Plenary Session 06: The Road Map to NSOAP Implementation

Speaker: Prof. Emmanuel Makasa, FCSECSA, MPH, MMed(Orth), MBChB, BSc.HB

The presentation highlighted several key points regarding the implementation of a national surgical healthcare policy. The overall goal is to achieve Universal Health Coverage, promote the health of the population, and ultimately contribute to the development of the country and continent. The government's role is essential, and Ministries of Health are encouraged to take ownership and lead the implementation process. To this end, strong governance is crucial, including the establishment of a director and technical teams committed to surgical care within the ministries of health. The involvement of additional partners should be limited to prevent external influence and maintain the power and agency of the ministries. Mobilization, coordination, and alignment of policies are emphasized, along with the importance of revising human resources for health establishments.

The national surgical policy should be aligned with existing plans in the respective ministries of health to avoid conflicts. The provision of surgical services should be established at all service levels, from primary to tertiary care. The Ministry of Health is encouraged to be the first to invest in surgical health systems, as initial investments can attract further support. Leveraging existing programs and entry points is recommended, with a focus on establishing surgical capacity for early biopsies at the district level. A robust health information management system is also essential for successful implementation as well as monitoring and evaluation efforts. The implementation should be evidence-based, contextualized, and consider prevention and rehabilitation aspects, including community education.

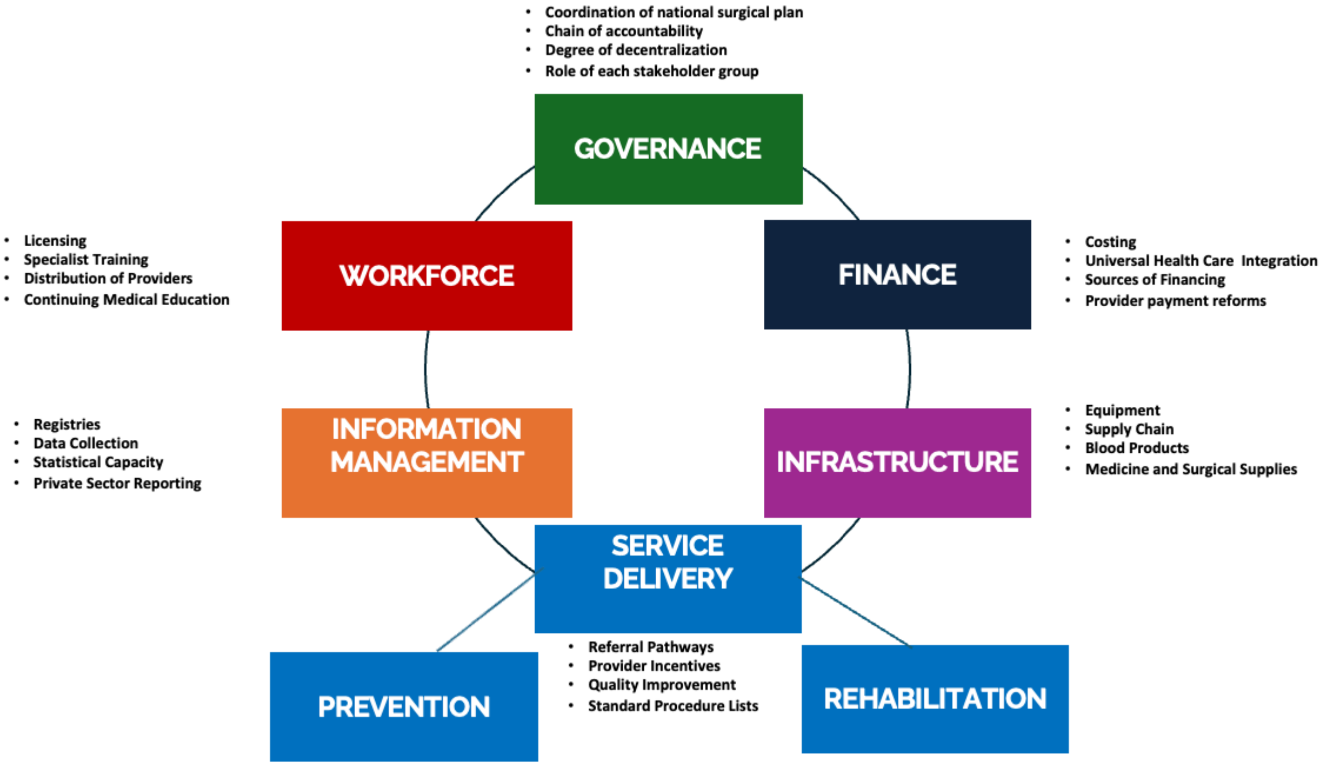

Fig. Pillars of National Surgical Plans

Accountability for the plans is crucial, with a focus on delivering quality services and avoiding unsafe surgeries. Sustainability is emphasized, and surgical access should be maintained regardless of changes in leadership. Any efforts to develop a national policy should be inclusive and decentralized, involving departments beyond medicine and expanding coverage to rural areas. The demand side perspective should be considered, allowing suggestions from patients and community members rather than making assumptions on their behalf. The development process of the policy should be guided by strong national government leadership and ownership.

Plenary Session 07: Roles of partners (Local and Global) in NSOAP development

Speaker: Robert Riviello, MD, MPH

Dr. Riviello's presentation on the role of partners in NSOAP Development started with a quote by the late Dr. Paul Farmer: " *With rare exceptions, all of your most important achievements on this planet will come from working with others—or, in a word, partnership."*. The session explored how different types of internal and external partnerships have helped facilitate the process of surgical healthcare planning in various ways. It was noted partners should not be the primary drivers of NSOAP development instead should be led by the national ministries. However, they play a critical role in supporting the development, implementation, monitoring, and evaluation of NSOAPs. Below are examples of the stakeholders that have made significant contributions to

NSOAPs through partnerships including academic Programs/ Institutions, Non-governmental Organizations, International organizations.

Examples of academic Programs/ Institutions

- **The Program in Global Surgery in Social Change (PGSSC)** at Harvard Medical School has a website with various global surgery resources, including all NSOAP documents.
- **McGill University** played a role in supporting Francophone countries in their NSOAPs development, collaborating with institutions like the West African College of Surgeons.
- **University of Witwatersrand: WitsSurg**
- **University of Global Health Equity** has a Center for Equity in Global Surgery (CEGS) that is starting a Master of Global Health Delivery program in Global Surgery. The center also offers a global surgery elective course for all MGHD students at the school in collaboration with Duke University. In addition, UGHE has multiple programs beneficial to global health professionals, including Executive Leadership training.

Non-governmental Organizations

- **Smile Train:** supported in setting up the conference and played a key role in Nigeria's NSOAPs development.
- **Operation Smile**: Supporting efforts in Madagascar, partnered with UGHE to develop and deliver a Global Surgery Advocacy Fellowship.
- **International, regional, and local professional societies** play important roles in advocacy, training, and resource provision. (E.g., World Federation of Societies of Anesthesiology, COSECSA, Rwanda Surgical Society)

International organizations

- Global Surgery Foundation and UNITAR
- Word Health Organization

Less explored partnerships:

- **Student Societies**: Particularly can help in advocacy and creating digital content
- **Funding Organizations**: World Bank, Monetary Fund, Gates Foundation
- **Patients and communities** to identify what is demanded and what matters to them and ensure the inclusion of all perspectives.

Partnership comes in various forms, offering different types of support. This includes technical support, resources including human resources, training in policy and advocacy, and knowledge resources. Regardless of the entities involved, all partnerships need to be built on trust, competency, reliability, transparency, and common ground if we are to achieve equitable access to surgical care.

Plenary Session 08: Funding in Global Surgery

Speaker: Prof. Emmanuel Ameh, MBBS, FWACS, FACS

The presentation centered around global surgery funding needs, particularly in Africa, how to strengthen current efforts, and highlighted potential opportunities for sustainable funding.

NSOAPs cost a small fraction (<1%) of the Health Sector Strategic Plan (HSSP) for countries with costed NSOAPs. Despite this, there exists a significant challenge in securing funds to expand the implementation of NSOAPs in other countries. The main reason for this is that many countries are simply not committing enough of their budget for healthcare, resulting in surgical care competing for funding. Contrary to the commitments countries made in the 2001 Abuja Declaration to allocate 15 % of the annual budget to healthcare, many have yet to reach the benchmark, with the majority allocating less than 10%. Furthermore, Africa heavily relies on external funding and out-of-pocket expenditure, leading to financial challenges and limited access to healthcare. Relying solely on external funding is deemed unsustainable due to the potential discontinuation/reduction of funding, priority misalignment with funders, and the cycle of dependence it creates.

To address these issues, there is a need to generate incremental domestic funding where external funding should serve primarily a supportive role. This will be a progressive process that can be done with regional commitment and collaboration. To achieve this, generating sustainable funding with a coordinated, realistic, and achievable framework is essential.

Suggestions for sustainable financing include advocating for increased government budget allocation, creating reliable community-based health insurance, leveraging private sector financing through partnerships, implementing taxation measures, and encouraging regional investments. Additionally, accessing external funding requires innovative approaches such as aligning surgical programs with existing program-based initiatives, incorporating research-based training, utilizing non-surgical healthcare funding, and leveraging partnerships with organizations focused on social innovation. The importance of collaboration, advocacy, and persistence in seeking funding opportunities was emphasized.

Despite challenges such as limited domestic resources, competing policies and needs of non-health sectors, and weak financial management. The goal is to collaborate rather than compete in the pursuit of funding while remaining innovative and determined to generate sustainable financial support for surgical healthcare.

Plenary Session 07: Advocacy in Global Surgery

Speaker: Justina Seyi-Olajide, MBBS, FWACS

Advocacy serves as a method to seek support for a certain cause, and in this context, the cause is to enhance surgical care for the population. We particularly need advocacy in surgical care as it is a field that has been neglected within the public health space in LMICs. Surgical care is considered too expensive and elitist, with much of the attention focusing on tertiary care, where most of the population can't easily access the service. Furthermore, there have been limited investment in surgical care with increased morbidity and mortality, long-term disability, and impoverishment of the population from surgical conditions. In order to advocate for this, one needs to have passion, compassion, and commitment to the cause and have a desire to change the status quo to shift from where we are to where we want to be.

The target of our advocacy efforts should be to expand access to safe surgical healthcare for the entire population, improve outcomes, and reduce the physical and socio-economic suffering of patients from surgical conditions. The advocacy cycle is an effective tool with key steps to achieve this.

Understanding: Initially, having a good understanding of the issue we are advocating for is very important. When we look at the burden of surgical disease, 5 billion people out of 7 billion worldwide, as of 2015, did not have access to safe surgical, obstetrics, and anesthesia care. This is a staggering number. In addition, advocating about the effects and consequences of lack of surgical care, such as increased complications, mortality, disability, and poverty, makes it a compelling topic for people to engage and support the cause.

Evidence-based Information: Data is a powerful tool for a convincing advocacy initiative, which we are lacking in most LMICs. Globally, there already exists compelling data on access, workforce, surgical volume, catastrophic healthcare expenditure, and cost-effectiveness around surgical care. 70% of the world's population lacks access to safe and affordable surgical care. Among them, 60% (3 billion) live in LMICs, 1.7 million are children (70% of the world's children), and out of this, 1.5 billion (88%) of the children reside in LMICs. Enhancing how we communicate these data using visuals such as imagery and graphs is very important. While global data is valuable, it is essential to have country-specific information. We must actively work toward generating the needed data if it is not already available in our respective nations.

The messaging: Having the right messaging is key in advocacy. The appropriate message varies based on the specific stakeholders you are addressing, whether they are policymakers, funders, or community members. Engaging individuals who are experts at this rather than surgeons/clinicians is crucial to tailor the message effectively for the intended audience.

Furthermore, using attractive messaging points is important and can be used to strengthen society's contribution. For example, when advocating for children’s surgery and cancer care, we can point out the different economic and social benefits to the child, the family, the community, and the country by investing in childhood cancer care.

Partners: It's important to seek and establish connections with relevant partners for the right messaging, to have the appropriate deployment platform, and to access key stakeholders.

Deployment: Having an advocacy toolkit that can be applied to different target audiences is essential. Advocacy initiatives can be deployed through various platforms, including channels like social and mainstream media, as well as through informal settings like community gatherings.

Evaluate and Innovate: It is important to periodically evaluate the impact of an advocacy initiative. We should check where the goals were achieved, what the challenges were and how they can be addressed, how to strengthen what is working well and change/eliminate what is not working well and introduce innovations to improve advocacy strategies.

Role of other Stakeholders: We need to consider partnerships with other stakeholders, including the population, communities, patients and their families, and celebrities, with their strong social media presence, all play a critical role in enhancing advocacy efforts.

In conclusion, it is imperative to start to think of surgery as a public health issue that impacts the entire population, which we can contribute to improve by harnessing advocacy as a tool.

Day 2: Breakout Sessions

Table 2: Day two breakout session groups

	Breakout Group Focus	Breakout Discussant Countries[TS1]
1	**What changes are required at the Ministries of Health level to implement significant surgical systems strengthening?**	Ethiopia, Namibia, Sierra Leone, Somalia, South Sudan, Tanzania, Zambia, Zimbabwe
2	**What should be the role of other ministries and arms of government in the design and implementation of National Surgical Policies?**	Ethiopia, Namibia, Sierra Leone, Somalia, South Sudan, Tanzania, Zambia, Zimbabwe
3	**What should Ministries of Health do within their ministry to support and sustain progress in National Surgical Plan implementation?**	Angola, Burkina Faso, Cabo Verde, Central African Republic, Chad, Comoros, Congo, Cote D'Ivoire, Egypt, Ethiopia, Equatorial Guinea, Gambia, Ghana, Guinea Bissau, Kenya, Lesotho, Liberia, Madagascar, Malawi, Mauritania, Namibia, Niger, Nigeria, Rwanda, Seychelles, Sierra Leone, Somalia, Somaliland, South Sudan, Tanzania, Togo, Zambia, Zimbabwe
4	**How can governments mobilize and coordinate efforts of local partners to support the design and implementation of National Surgical Plans/Policies?**	Angola, Cabo Verde, Equatorial Guinea, Kenya, Somaliland, Mauritania, Zambia

5	**What should the roles of inter-governmental bodies in the planning and implementation of National Surgical Plans/ Policies?**	Angola, Burkina Faso, Burundi, Central African Republic, Chad, Comoros, Cote D'Ivoire, Egypt, Gambia, Ghana, Guinea Bissau, Kenya, Lesotho, Liberia, Malawi, Niger, Nigeria, Rwanda, Togo,
6	**How can ministries integrate funding of surgical and anesthesia care to other existing healthcare systems in a country?**	Angola, Egypt, Gambia, Ghana, Guinea Bissau, Kenya, Lesotho, Malawi, Nigeria
7	**What do you suggest as the way forward?**	Angola, Burundi, Cabo Verde, Central African Republic, Chad, Comoros, Congo, Côte d'Ivoire, Egypt, Equatorial Guinea, Ethiopia, Gambia, Ghana, Guinea-Bissau, Kenya, Lesotho, Liberia, Madagascar, Malawi, Namibia, Niger, Nigeria, Rwanda, Seychelles, Sierra Leone, Somalia, Somaliland, Tanzania, Togo, Uganda, Zambia, Zimbabwe

Discussion and Report

1. **What changes are required at the Ministries of Health level to implement significant surgical systems strengthening?**

Delegates highlighted the importance of identifying the surgical disease burden in a country and building a comprehensive surgical roadmap that informs policy/plan development. This process is crucial for proper resource allocation at all levels in the healthcare system. Strengthening of data collection systems, with clear indicators including service quality assessments, and integration into the national database such as Demographic Health Surveys (DHS) were underscored. Rather than having a desk within MoH for National Surgical Healthcare Plan/Policy development and implementation establishing a specialized a unit or directorate was recommended. Furthermore, it was also suggested to formulate an inter-ministerial task force to play an advisory role and district level committees to ensure practice implementation of the

policies and plans. Delegates stressed the importance of multi-sectoral collaboration and advocacy at all levels inclusive of community engagement.

Proper budget allocation for surgical system strengthening was mentioned as a necessity to reduce dependency on external funding. Improving infrastructure, medical equipment, and supply chain management, and having an effective monitoring and evaluation mechanism that feed into strong feedback loops can significantly improve surgical serviced delivery. Another Important suggestion raised by delegates is Integrating NSOAPs into other existing plans such as health strategic plans and national development plans.

2. **What should be the role of other ministries and arms of government in the design and implementation of National Surgical Policies?**

The role of other ministries and governmental bodies, including parliament and the cabinet, in the design and implementation of National Surgical, Obstetric, and Anesthesia Plans (NSOAPs) was discussed with a focus on collaboration and communication. Suggestions included scheduling regular inter-ministry meetings to discuss ongoing projects and plan future steps, emphasizing a ministerial social pillar to enhance cooperation. Additionally, there was a proposal to organize a forum where countries could share best practices, fostering mutual learning and collaboration. To facilitate continuous evaluation and knowledge-sharing, the idea of setting up a committee that meets more frequently was put forward. These recommendations underscored the importance of inter-sectoral engagement and coordinated efforts for the successful implementation of NSOAPs.

3. **What should Ministries of Health do within their ministry to support and sustain progress in National Surgical Plan implementation?**

Delegates emphasized several key actions that Ministers of Health should undertake within their ministries to support and sustain progress in NSOAP implementation. Firstly, there was a call for the integration of surgical care policies into the national policy for development, ensuring their alignment with overarching national strategies. To ensure continuity, mechanisms should be in please to minimize policy changes with a change in leadership within MoHs. Obtaining buy-in from the country's president and governmental oversight and enforcement to ensure adherence to these policies was identified as a crucial step, emphasizing the need for high-level support.

Financial commitment through budget allocation, with potential support from international organizations such as WHO, IMF, and the World Bank, was deemed essential. Inclusivity was emphasized, stressing the involvement of all stakeholders, including ethics, research, and statistics commissions, as well as other relevant ministries. Sharing micro plans with the WHO

representative in each country was proposed to enhance collaboration. Assigning a focal person for surgical health care within the Ministry of Health for each country and ensuring the alignment of NSOAP policies with existing policies were mentioned as integral steps toward sustained progress in NSOAP implementation.

4. **How can governments mobilize and coordinate efforts of local partners to support the design and implementation of National Surgical Plans/Policies?**

Delegates suggested for governments to engage in bilateral collaborations with Non-Governmental Organizations (NGO) and replicate successful strategies from other countries while adapting them to the local context. They also highlighted the importance of identifying key stakeholders within the country and convening them to share experiences and insights, fostering a collaborative environment and drawing inspiration from successful initiatives in the region.

5. **What should the roles of inter-governmental bodies in the planning and implementation of National Surgical Plans/ Policies?**

Delegates emphasized the crucial roles of intergovernmental bodies in the planning and implementation of National Surgical Policy/plan. They highlighted the importance of these bodies in monitoring and evaluating the progress of Plan/Policies progress. They mentioned the need for collaboration between postgraduate colleges and ministries of health to provide support with resources. They further recommended sharing of technical skills among the college of surgeons in different countries and integrating training programs and research, as well as focusing on capacity building. Delegates also emphasized these bodies have a critical role to play in regional and national coordination of initiatives to improve surgical care and provide external validity and ownership.

6. **How can ministries integrate funding of surgical and anesthesia care to other existing healthcare systems in a country?**

To integrate funding for surgical and anesthesia care into existing healthcare systems, several key strategies were suggested. Firstly, there was an emphasis on the creation of a dedicated budget line specifically allocated by the Ministry of Finance. This designated funding channel would ensure a targeted approach to surgical, obstetrics and anesthesia care. Delegates highlighted the importance of budgeting for surgical care at all levels, starting from the lowest level of healthcare institutions. Granting institutions at lower levels autonomy to develop their own budgets would enable tailored financial plans that address specific needs. Additionally, it was recommended that the budget should be dynamic, with ongoing monitoring and evaluation mechanisms in place to facilitate necessary adjustments based on evolving requirements.

7. **What do you suggest as the way forward?**

Delegates outlined a comprehensive set of recommendations for the way forward in advancing surgical care in Africa. Firstly, there was a strong call for national ownership of surgical system strengthening, emphasizing the need for a well-defined framework for effective implementation. Providing a platform to share experiences and consolidate best practices among African countries was highlighted as a crucial step. Advocacy for surgical care as a public health concern and for promoting integrating into national and regional health agendas was highlighted. Capacity building programs for healthcare professionals at all levels, encompassing both short-term and long-term training, were recommended to enhance skills and expertise.
Strengthening monitoring and evaluation systems to track progress and ensure quality improvement emerged as a key focus. Exploring innovative financing mechanisms and engaging with the private sector were suggested to mobilize resources for surgical system strengthening. Harmonizing curricula and standards for surgical training across African countries was proposed to ensure consistent quality and competency among healthcare professionals. Furthermore, delegates encouraged the standardization of surgical practices and guidelines aimed at improving patient outcomes and safety.

PASHeF Consensus Process

The model ensured the process was done with inclusive and open participation from all the delegates where multiple opportunities were given provide feedback, seek clarification, express agreement, or reservations with further elaborations. The consensus document underwent four iterations, it was presented visually to review the points raised line by line where the document was being edited and updated real-time based on the response and feedback from delegates. This was done until a unanimous agreement was reached by all the delegates on the final product of the document consolidating the PASHeF 2023 Consensus Statement.

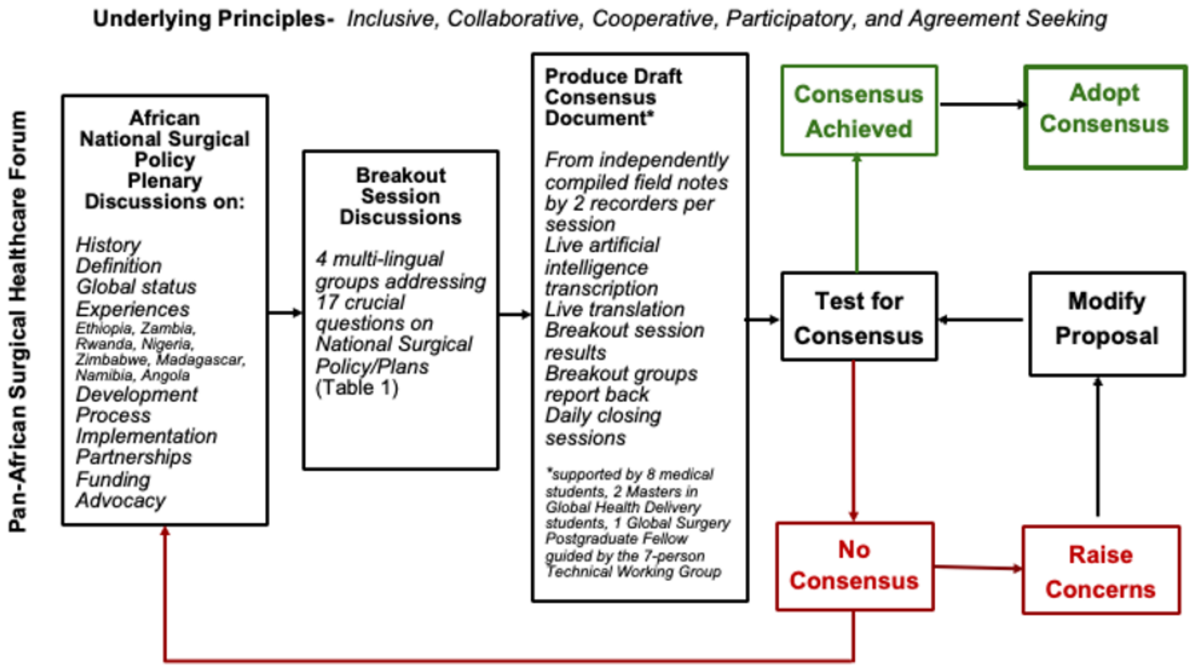

Fig. The PASHeF Consensus Process

PASHeF Consensus Statement

The statement consists of 50 points grouped under 15 categories as stated below.

1. Relevance

Key words: "Relevant"

2. Importance

Key words: "National Health Systems Strengthening", "Important" "Implementation"

3. Pitfalls

Key words: "Implementation" "infrastructure" "equipment" "supply chain" "research" "data" "change"

4. National Policy Integration

Key words: "Implementation" "infrastructure" "equipment" "supply chain" "research" "data" "change"

5. Information and Data

Key words: Data" "National Health Information Systems" "DHIS" "Monitoring and Evaluation" "Indicators"

6. Opportunities

*Key words: "*Sharing" "Partnerships" "Youth" "Communities"

7. Barriers

Key words: "Financing" "Political will" "Expertise" "Training" "Motivation" "Resources" "Equipment"

8. Proposed Steps of Development

Key words: "Appropriate" "Community" "Prevention" "Rehabilitation" "Capacity building" "Sustainability"

9. Monitoring and Evaluation

Key words: "Monitoring and evaluation" "Regional" "Quality Assurance"

10. MOH: Leadership

Key words: "Directorate" "Department" "Continuity" "Inter-ministerial task force" "Technical Working Group"

11. MOH: Resources

Key words: "Budget" "Essential drug list" "Innovative financing", "Private sector"

12. Stakeholders and Partners

Key words: "Ministry of Health" "Ministry of Finance" "Partnership" "Community" "Academia" "Industry" "Military" "Funding agencies" "Regional"

13. Advocacy

Key words: "Stakeholders" "Celebrities" "Grassroots advocacy" "Media"

14. Regional Harmonization

Key words: "regional"

15. Way forward for PASHeF (Structure and Logistics)

Key words: "Annual" "Governance"

Way Forward

The way forward for the PASHeF was discussed amongst delegates, with an agreement to prioritize the transmission of a consensus statement, under the directive of the Honorable Minister of Health, Republic of Rwanda, via formal letter to African Ministries of Health. The letter to ministers (translated to official language of the specific country) will encompass expressions of appreciation, vital information about the conference, the consensus statement, and invitation for a follow up meeting with the ministers. Additionally, there was a proposal for the establishment of PASHeF as an annual platform for Ministries of Health (MoHs) to take ownership and engage with relevant themes each year. The platform is designed to facilitate the sharing of experiences, requests for technical exchange and support, engagement with global partners, and the invitation of other global partners to the annual sessions. The plan includes a rotation of host countries, the appointment of a focal person from each African country represented at PASHeF, the development of an inclusive governance system, documentation of meeting outputs for stakeholder sharing, and support from the secretariat to implement these initiatives. It was emphasized that each MoH participating in PASHeF should designate a focal person. These individuals would collaborate with the PASHeF secretariat, currently set to be based at the

University of Global Health Equity (UGHE) to guide and support the effective implementation of the vision and mission of PASHeF.

Closing Remark

In his closing remarks, Prof. Abebe Bekele extended a heartfelt message to all delegates, urging them to disseminate the consensus statement within their respective ministries. He officially concluded the forum by expressing sincere gratitude to the Rwandan government and the Minister of Health for graciously hosting the PASHeF event. Prof. Abebe further acknowledged Smile Train and its leadership for their invaluable collaboration in supporting the event, along with appreciation for the core technical members and the dedicated coordinating team from the Center for Equity in Global Surgery (CEGS) at UGHE.

Appendices

Appendix (S3 Appendix)

PASHeF 2023 Consensus Statement (available online with PASHeF Consensus Article)
