# Supplementary material for: The Pan-African Surgical Healthcare Forum: An African qualitative consensus propagating continental national surgical healthcare policies and plans
Source: PLOS Glob Public Health. 2024 Nov 12;4(11):e0003635. doi: 10.1371/journal.pgph.0003635 (PMC11556714; doi:10.1371/journal.pgph.0003635)
Supplement: S5 Appendix — (PDF) [file pgph.0003635.s006.pdf]

S5 Appendix. Reflexivity Statement

1. **How does this study address local research and policy priorities?**

This research was specifically designed by local (Low-and Middle-Income Country) partners to assess and address equity in access to surgical care. Over 76% of the continent's population do not live in countries with actionable national surgical healthcare policies. Even in countries with such policies or plans, there are gaps in dissemination, funding, and implementation This forum is a local attempt by African Ministries of Health to address this free from outside influence. This is a key priority of the Rwanda Ministry of Health, the University of Global Health Equity and its Centre for Equity in Global Surgery, and the Forum Technical Committee's home institutions (South African Development Community's University of Witwatersrand Regional Collaboration Centre for Surgical Healthcare Improvement all from low-income country contexts.

2. **How were local researchers involved in study design?**

AB, EA, BA, AM, FN, and JS are Low- and Middle- Income Country (LMIC) surgical educators and researchers who have extensive experience in leading and organizing international Global Health educational collaborations and surgical health policy. They worked closely with RR, a High-Income Country (HIC) colleague and friend who has worked for several years in a LMIC in his capacity as the co-Chair of an LMIC Center for equity in Global Surgery, and with NK, vice president and Africa coordinator of a not-for-profit to design the consensus process and methodology.

3. **How has funding been used to support the local research team?**

This forum entailed logistics of organizing the PASHeF meeting. Funding for the Forum was provided by Smile Train, and co-sponsored by the University of Global Health Equity. However, the investigators were not paid for research and no funding was provided for publication.

4. **How are research staff who conducted data collection acknowledged?**

All those who conducted data collection are included as collaborators and authors and have been acknowledged openly during the PASHeF forum as members of the logistics committee. In addition to data collection, they participated in the process of Forum didactics delivery, evaluation, analysis, writing, and editing.

5. **Do all members of the research partnership have access to study data?**

Yes, all members of the partnership have access to study data.

6. **How was data used to develop analytical skills within the partnership?**

Team members worked together to leverage the variety of skills sets. BA, AB, BF, JS, NO, RR, EM, and EA analysed the data. Presentation of the analysis was reviewed and refined by the senior researchers. In so doing, BA and BF's analytical skills were improved.

7. **How have research partners collaborated in interpreting study data?**

Study data has been interpreted collaboratively by BA, BF, JS, NO, RR, EM, and EA. Results have been discussed verbally and in writing to arrive at appropriate interpretations. The entire Forum (all

collaborating authors) collaboratively looked at data through multiple sessions to arrive at consensus.

8. How were research partners supported to develop writing skills?

Early career researchers (BA and BF) on the authorship team were supported by senior global health academics to develop and refine their writing skills. Multiple reviews and suggestions were made by senior members of the team to improve the early drafts. RR, and AB support BA's writing beyond this project, and BA supports BF's scientific writing development.

9. How will research products be shared to address local needs?

The PASHeF consensus statements in French, Spanish, Portuguese, and English have been disseminated widely to African Ministers of Health to support delivery of surgical healthcare. This publication will be the charter of sorts for African national surgical planning. This publication will also serve to enhance surgical advocacy for surgical healthcare. We intend that this article will be published as open access. Our post-publication dissemination plan will include presentation at local and regional global health and global surgery conferences. We hope to engage with leaders in global health, and surgical education who are involved in educational collaborations, and with journalists, both based in HICs and LMICs. The records will be made available to the local university libraries, to support iterations of future courses.

10. How is the leadership, contribution, and ownership of this work by LMIC researchers recognised within the authorship?

Authors EM, EA, and AB (from LMICs) were key in the senior authorship team in developing this manuscript, and their contribution has been recognised as joint senior/last authors.
BA's contribution to the work has been recognised as he is acknowledged as the first and corresponding author. The authorship author was deliberate. Authors from and based in the global south are both senior authors and first authors.

11. **How have early career researchers across the partnership been included within the authorship team?**

We have included early career researchers (BA and BF) within the authorship team. They participated in all parts of the programme and were deeply embedded in the forum. They led the work hands-on, collected data, performed literature review and evidence synthesis, and developed drafts, and contributed to reviews. BA and BF are based in an LIC.

12. How has gender balance been addressed within the authorship?

Six authors are male (BA, FN, RR, EM, EA and AB) and three authors are female (JS, BF, NO). After BA, who contributed significantly to the work, the 2 female authors JS and BA have been acknowledged as second and third authors (higher acknowledgements) before any other male author reflecting balance based on their contributions. We acknowledge that more female authors would have contributed to improve the balance. Additional female faculty has been hired at the key partner LMIC institution, which is committed to gender balance, although among surgical trainees in the country who will be trained into the employment pool, only 1 in 15 are female. BA, AB, BF, and RR's LMIC institution characteristically admits more female than male medical students in a ratio of 2:1.

13. How has the project contributed to training of LMIC researchers?

BA as an LMIC researcher was mentored by both HIC and LMIC senior researchers (AB, RR, EM, JO, EA) through stages of the study. Since the forum, BF has been supported by RR and BA into an academic research fellowship position. AB and EA based in LMICs (East and West Africa respectively) are especially senior researchers and have guided the team. EM is a very senior policy expert having lived and worked in Geneva with the UN as a special envoy. He has trained the rest of the team in dynamics and relationships with diplomats, envoys, WHO, and ministries of Health.

14. How has the project contributed to improvements in local infrastructure?

This project has directly contributed to improvements in local educational infrastructure by driving improvements in national surgical healthcare plans/policy. The Forum is active on WhatsApp and countries are progressively being supported.

15. What safeguarding procedures were used to protect local study participants and researchers?

Both the Rwanda Ministry of Health, UGHE, and Smile Train have stringent safeguarding university policies and channels available to students and staff through which complaints and assistance could be freely and easily sought in case of any harassment. Power dynamics between researchers were directly addressed. Participation in the Forum was completely voluntary, and all data was collected voluntarily from forum participants (ministry delegates) who had signed. The process of consensus was open and transparent.
