# Supplementary material for: The Pan-African Surgical Healthcare Forum: An African qualitative consensus propagating continental national surgical healthcare policies and plans
Source: PLOS Glob Public Health. 2024 Nov 12;4(11):e0003635. doi: 10.1371/journal.pgph.0003635 (PMC11556714; doi:10.1371/journal.pgph.0003635)
Supplement: Attachment — Submitted filename: Response to Reviewers.docx [file pgph.0003635.s007.docx]

Response to Reviewers

Thank you for your kind review and suggestions to improve this manuscript. All suggestions have been applied to improve the work.

| Comment | Response | Action | Location |
| --- | --- | --- | --- |
| **Journal and Editorial Comments**- Oliver Ian Tolentino (August 15, 2024)   1. Please provide a detailed online Financial Disclosure statement. This is published with the article. It must therefore be completed in full sentences and contain the exact wording you wish to be published. If you did not receive any funding for this study, please simply state: “The authors received no specific funding for this work.” | This has been done. We did not receive any funding fro this study. | The authors received no specific funding for this work. | Online Financial Disclosure statement |
| 2. Some material included in your submission may be copyrighted. Potential Copyright Issues:   Figure 5, S3 Appendix and S4 Appendix contain branding/a logo. We are not permitted to publish this under our CC-BY 4.0 license, even with permission. We ask that you please remove or replace it. | PASHeF and ***all*** other Logos have been removed as requested. | PASHeF and *all* other Logos have been removed from Figure 4, S3 Appendix and S4 Appendix | Figure 5, S3 Appendix cover and S4 Appendix |
| 3. Figures 3, 4 and 5: Please confirm whether you drew the images / clip-art within the figure panels by hand. If you did not draw the images, please provide (a) a link to the source of the images or icons and their license / terms of use; or (b) written permission from the copyright holder to publish the images or icons under our CC-BY 4.0 license. Alternatively, you may replace the images with open source alternatives. | All images in Figures 3,4,5 have been drawn by the first author. Images/clip art within the figure panels are drawn by hand. | I confirm that all images within the figure panels (Figures 3, 4, and 5) have been drawn by hand. | Figures 3, 4, and 5 |
| 4. Figure 2: please (a) provide a direct link to the base layer of the map (i.e., the country or region border shape) and ensure this is also included in the figure legend; and (b) provide a link to the terms of use / license information for the base layer image or shapefile. We cannot publish proprietary or copyrighted maps (e.g. Google Maps, Mapquest) and the terms of use for your map base layer must be compatible with our CC-BY 4.0 license. Note: if you created the map in a software program like R or ArcGIS, please locate and indicate the source of the basemap shapefile onto which data has been plotted.  If your map was obtained from a copyrighted source please amend the figure so that the base map used is from an openly available source. Alternatively, please provide explicit written permission from the copyright holder granting you the right to publish the material under our CC-BY 4.0 license. | (a) Direct link to the base layer of the map (i.e., the country or region border shape) is <https://www.mapchart.net/africa.html> and this is provided within the figure legend. (b) A link to the terms of use/licence information for the base layer image or shapefile is included <https://www.mapchart.net/terms.html#licensing-maps> | (a) Direct link to the base layer of the map (i.e., the country or region border shape) is <https://www.mapchart.net/africa.html> and this is provided within the figure legend. (b) A link to the terms of use/licence information for the base layer image or shapefile <https://www.mapchart.net/terms.html#licensing-maps> | Figure 2 |
| Journal Comments (Earlier Review)  1. Please include a complete copy of PLOS’ questionnaire on inclusivity in global research in your revised manuscript. Our policy for research in this area aims to improve transparency in the reporting of research performed outside of researchers’ own country or community. The policy applies to researchers who have travelled to a different country to conduct research, research with Indigenous populations or their lands, and research on cultural artefacts. The questionnaire can also be requested at the journal’s discretion for any other submissions, even if these conditions are not met.  Please find more information on the policy and a link to download a blank copy of the questionnaire here: [https://journals.plos.org/globalpublichealth/s/best-practices-in-research-reporting](https://journals.plos.org/globalpublichealth/s/best-practices-in-research-reporting). Please upload a completed version of your questionnaire as Supporting Information when you resubmit your manuscript. | PLOS’ questionnaire on inclusivity in global research Included | Plos Inclusivity in global research checklist included  Inclusivity in global research section included  “Members of the local communities guided the aims, were integral to the methodology and anticipated outcomes as local participants at the Pan African surgical Healthcare Forum. This was an inclusive African consensus. Additional information regarding the ethical, cultural, and scientific considerations specific to inclusivity in global research is included in the Supporting Information (S6 Checklist).” | Lines 162-167  Line 703  Supplement 6  Checklist |
| 2. Please review your reference list to ensure that it is complete and correct | Reviewed | list is complete and correct | Page 34  References |
| **Reviewer #1**: |  |  |  |
| **Reviewer #1**: I would like to congratulate the authors on an outstanding report on this effort.  I have a few questions for further clarification-  Did any discussion of vulnerable populations such as pediatrics come up or was the conversation broader?. | Discussions of vulnerable populations, particularly pediatrics came up particularly in the case studies from Nigeria and Madagascar National Surgical Healthcare Plan and in discussions on advocacy in Global Surgery. The discussions were focused on the high proportion of the African population that is the pediatric population; the high burden of pediatric surgical disease; and 2 case studies on the approach a few African countries have chosen to acknowledge this in their surgical plans. | This is included in the Appendix 4, and a statement highlighting the case studies with emphasis on pediatric vulnerable populations has been added to the text.  “Discussions about vulnerable populations such as pediatrics stemmed from national surgical planning case studies from Nigeria and Madagascar (see S4 Appendix. Proceedings and empirical data), and participants acknowledged the high proportion of children in Africa as a peculiar consideration in planning for surgical care on the continent.”  This has already been highlighted in the Nigeria and Madagascar case studies (S4 Appendix). | Lines 312-315;  Lines 513-514 |
| Indicators were noted very generally - where there any comments on what LCoGS or other indicators may be considered? Again the discussion may not have been in this detail but surely an important next step will be evaluating systems | While participants indicated that countries should have a clear surgical care monitoring and evaluation tools and sets of indicators, and that peer accountability based on these is crucial, there was no attempt at pursuing consensus geared at any specific selected regional indicators. Identifying cross-cutting indicators will be a crucial next step in evaluating systems and will be the focus of future discussions. Countries envision that specific indicators can be collaboratively assessed for peer accountability, and as noted by the reviewer, indicators were only noted very generally. | This has been noted for future discussion in future PASHeF meetings by technocrats.  Included in the limitations/discussion:  “While participants indicated that countries should have clear surgical care monitoring and evaluation tools and sets of indicators, and that peer accountability based on these is crucial, there was no attempt at pursuing consensus geared at any specific selected regional indicators. Identifying cross-cutting indicators will be a crucial next step in evaluating systems and will be the focus of future discussions. Countries envision that specific indicators can be collaboratively assessed for peer accountability, and this will be the focus of future discussions for PASHeF.” | Line 555-561  See 527-533 |
| Were there any specific lessons/success stories or challenges from any participating country that came up during the meeting? It would be useful to have these as small case studies or included in the report in some fashion. | Success stories, specific lessons and challenges from Ethiopia (*Elubabor Buno)*, Zambia (*Christopher Chanda), Nigeria (Bitrus Oghoghorie Deborah), Zimbabwe* (*Shingai Nyaguse), Madagascar (Rado Razafimahatratra), Namibia (Francina Marukuavi Ngakuzevi), Rwanda (Parfait Uwaliraye) and Angola. These have been included in S4 Appendix.* | These case studies have been highlighted S4 Appendix.  **Case Study 1: Experience from Ethiopia**  **Case Study 2: Experience from Zambia**  **Case Study 3: Experience from Nigeria**  **Case Study 4: Experience from Zimbabwe**  **Case Study 5: Experience from Madagascar**  **Case Study 6: Experience from Namibia**  **Case Study 7: Experience from Rwanda**  **Case Study 8: Experience of Angola**  Case studies highlighting success stories, lessons and challenges from all 8 countries are mentioned in the main manuscript, and included in detail in Appendix 4 | Case Studies included (Highlighted headings in S4 Appendix) on page 13-32 and 41-42  Line 260-265 |
| In the M&E section on NSOAPs, why do African MOHs feel these are inadequate? What do propose differently? How would that be better? | Country delegates felt that internal monitoring and evaluation within countries was not sufficient and proposed regional action with a peer monitoring and evaluation system. They thus proposed the creation of a Pan-African/regional Surgical Healthcare Policy monitoring system composed of member states to follow up the continental progress. They also suggested that monitoring and evaluation should be aligned with Quality Assurance. While monitoring and evaluation was seen as more focused on indicators and process or outcome measures, quality assurance was seen as more proactive, less focused on reprimands while ensuring that national surgical healthcare programs meet the needs and expectations of populations.   1. Building regional peer monitoring systems for national surgical healthcare plans. 2. Strengthening data collection systems for M and E and integrating these into national databases such as Demographic Health Surveys 3. Establishing a specialized unit or directorate focused on National Surgical Healthcare Plan/Policy development and implementation (including M and E and Quality Assurance)rather than having only a desk within the MoH. 4. Increase the frequency of evaluation. Delegates suggested that countries move beyond endline evaluations to include consistent midpoint evaluations prior to endline (e.g. Rwanda). | Added to the M&E section.  “Country delegates stated that internal monitoring and evaluation within countries was not sufficient and proposed regional action with a peer monitoring and evaluation system. They proposed the creation of a Pan-African/regional Surgical Healthcare Policy monitoring system composed of member states to follow up continental progress in surgical care. They also suggested that monitoring and evaluation should be aligned with Quality Assurance. While monitoring and evaluation was seen as more focused on indicators and process or outcome measures, quality assurance was seen as more proactive and less focused on reprimands while ensuring that national surgical healthcare programs meet the needs and expectations of population throughout the duration of the national surgical plan implementation. Monitoring and evaluation could thus be improved by regional peer monitoring systems, strengthening data collection systems and establishing dedicated specialized units or directorates for surgical healthcare Monitoring and Evaluation. Mid-term evaluation also need to be carried out as in the Rwanda case study.” | Monitoring and evaluation of plan/policy implementation section  Lines 427-439 |
| Line 507, I'm not sure I understand what "supporting similar internally led processes" refers to. Also in the next sentence, there is reference to academic resources but one usually would not consider "staff, stuff, space, systems" academic resources. These two minor points should be considered. | Clarified. | “by supporting similar internally led processes” changed to “by supporting more locally led, horizontal, capacity building programs.” | Changed in Line 543 |
| Lastly there are few scattered punctuation errors - missing apostrophes and lack of capitalization etc. | Writing was passed through Grammarly and noted punctuation errors corrected. | Punctuation errors identified and corrected |  |
| Thank you for the opportunity to review this work and give constructive feedback. Congrats once again to the authors for a truly inspiring effort. | Thank you very much |  |  |
| Reviewer #2 |  |  |  |
| Reviewer #2: 1. With the use of the constructivist method, use of 'key words' in the coding to facilitate comparative analysis of the observations and declarations would be important to denote in the Appendix. This was unclear in Appendix 4. | Key words are included in Appendix 4. | Included in Appendix 4.  1. Relevance  *Key words: “Relevant”*  2. Importance  *Key words: “National Health Systems Strengthening”, “Important”*  3. Pitfalls  *Key words: “Implementation” “infrastructure” “equipment” “supply chain” “research” “data” “change”*  4. National Policy Integration  *Key words: “infrastructure” “equipment” “supply chain” “research” “data” “change” “Implementation”*  5. Information and Data  *Key words: Data” “National Health Information Systems” “DHIS” “Monitoring and Evaluation” “Indicators”*  6. Opportunities  *Key words: “*Sharing” “Partnerships” “Youth” “Communities”  7. Barriers  *Key words: “Financing” “Political will” “Expertise” “Training” “Motivation” “Resources” “Equipment”*  8. Proposed Steps of Development  *Key words: “Appropriate” “Community” “Prevention” “Rehabilitation” “Capacity building” “Sustainability”*  9. Monitoring and Evaluation  *Key words: “Monitoring and evaluation” “Regional” “Quality Assurance”*  10. MOH: Leadership  *Key words: “Directorate” “Department” “Continuity” “Inter-ministerial task force” “Technical Working Group”*  11. MOH: Resources  *Key words: “Budget” “Essential drug list” “Innovative financing”, “Private sector”*  12. Stakeholders and Partners  *Key words: “Ministry of Health” “Ministry of Finance” “Partnership” “Community” “Academia” “Industry” “Military” “Funding agencies” “Regional”*  13. Advocacy  *Key words: “Stakeholders” “Celebrities” “Grassroots advocacy” “Media”*  14. Regional Harmonization  *Key words: “regional”*  15. Way forward for PASHeF (Structure and Logistics)  *Key words: “Annual” “Governance”* | Supplement 4. Appendix. Page 54. |
| 2. Perhaps as a limitation, or an opportunity for further discussion, involvement of the established collegiate/ university surgical training programs as stakeholders may aid in percolating global surgery, as well as enhancing regional harmonisation. In conjunction with the respective Ministries of Health, these would serve as valuable avenues through which the respective countries would reach their doctor- population ratios stated in the NSOAPs, or facilitate channels for financial aid, act as centres for monitoring and evaluation of the policies once they would be rolled out. These already being present in some of the mentioned countries, should have representation/ be mentioned. | Thank you; this is very much acknowledged and included. Due to the limitation of time at the PASHeF 2023 meeting, this was not explored in detail and will be a future direction. In consensus statement 35, country delegates identified critical stakeholders in the process of Surgical  Healthcare Plan/Policy development: including professional societies and academia (e.g. COSECSA, WACS). | Included in the limitations.  “While discussion on the role of academic and other partners came up during plenary and was highlighted in consensus statement 35, further discussion is necessary given their crucial role in global surgery and PASHeF consensus implementation. For instance, the involvement of established African regional collegiate surgical training programs (particularly the West African College of Surgeons, the College of Surgeons of East Central and Southern Africa, national postgraduate medical colleges of surgery) and university surgical training programs as stakeholders will aid in enhancing regional harmonization and can serve as academic think-tanks for Ministries of Health. They are key avenues through which countries can reach appropriate doctor- population ratios stated in their surgical plans, channels for financial aid, and can provide for monitoring and evaluation and quality assurance of policies. The means of engagement and onboarding of partners with PASHeF must be outlined and discussed by member states in the future.” | Lines 561-572 |

Thank you for your kind review and improvement of this manuscript.

With gratitude,
